# Gene-Editing Technologies Paired With Viral Vectors for Translational Research Into Neurodegenerative Diseases

**DOI:** 10.3389/fnmol.2020.00148

**Published:** 2020-08-12

**Authors:** Joseph Edward Rittiner, Malik Moncalvo, Ornit Chiba-Falek, Boris Kantor

**Affiliations:** ^1^Department of Neurobiology, Duke University Medical Center, Durham, NC, United States; ^2^Viral Vector Core, Duke University Medical Center, Durham, NC, United States; ^3^Duke Center for Advanced Genomic Technologies, Durham, NC, United States; ^4^Department of Neurology, Division of Translational Brain Sciences, Duke University Medical Center, Durham, NC, United States; ^5^Center for Genomic and Computational Biology, Duke University Medical Center, Durham, NC, United States

**Keywords:** neurodegenarative diseases, AAV vectors, lentiviral (LV) vector, CRISPR-Cas 9 system, gene editing, epigenetics (DNA methylation, histone modifications)

## Abstract

Diseases of the central nervous system (CNS) have historically been among the most difficult to treat using conventional pharmacological approaches. This is due to a confluence of factors, including the limited regenerative capacity and overall complexity of the brain, problems associated with repeated drug administration, and difficulties delivering drugs across the blood-brain barrier (BBB). Viral-mediated gene transfer represents an attractive alternative for the delivery of therapeutic cargo to the nervous system. Crucially, it usually requires only a single injection, whether that be a gene replacement strategy for an inherited disorder or the delivery of a genome- or epigenome-modifying construct for treatment of CNS diseases and disorders. It is thus understandable that considerable effort has been put towards the development of improved vector systems for gene transfer into the CNS. Different viral vectors are of course tailored to their specific applications, but they generally should share several key properties. The ideal viral vector incorporates a high-packaging capacity, efficient gene transfer paired with robust and sustained expression, lack of oncogenicity, toxicity and pathogenicity, and scalable manufacturing for clinical applications. In this review, we will devote attention to viral vectors derived from human immunodeficiency virus type 1 (lentiviral vectors; LVs) and adeno-associated virus (AAVs). The high interest in these viral delivery systems vectors is due to: (i) robust delivery and long-lasting expression; (ii) efficient transduction into postmitotic cells, including the brain; (iii) low immunogenicity and toxicity; and (iv) compatibility with advanced manufacturing techniques. Here, we will outline basic aspects of LV and AAV biology, particularly focusing on approaches and techniques aiming to enhance viral safety. We will also allocate a significant portion of this review to the development and use of LVs and AAVs for delivery into the CNS, with a focus on the genome and epigenome-editing tools based on clustered regularly interspaced short palindromic repeats/CRISPR-associated protein 9 (CRISPR/Cas 9) and the development of novel strategies for the treatment of neurodegenerative diseases (NDDs).

## Introduction

As of this year, more than seven million Americans suffer from neurodegenerative disorders, with the majority of cases due to Alzheimer’s disease. By 2050, this number is projected to rise to nearly 14 million (Alzheimer’s Dementia, 2020). In addition to the lost quality of life, these increasingly prevalent conditions impose a major financial burden on our society. Alzheimer’s and other dementias will cost the U.S. economy an unbelievable $305 billion in 2020, with projected costs rising as high as $1.1 trillion/year by 2050 (Alzheimer’s Dementia, 2020). As such, effective preventive and therapeutic approaches are desperately needed. Unfortunately, current pharmacological treatments provide only temporary symptomatic relief (if that), without addressing the underlying causes.

Virus-mediated gene therapy, on the other hand, is a viable long-term strategy for the disease-modifying treatment of several neurological and neurodegenerative disorders. Traditionally, “gene therapy” has entailed the introduction of an entire gene, which either compensates for a malfunctioning gene or provides a new function to cells which allows them to better combat a disease state. Recently, however, researchers have gained the ability to introduce constructs that can edit the genome—or alter gene expression by modifying the epigenome—with astonishing precision and flexibility. These recent advances are primarily the result of engineering a bacterial defense system called clustered regularly interspaced short palindromic repeats (CRISPR), which we will review in-depth. Indeed, the appealing prospect of treating diseases at the root of their cause has led to considerable efforts toward the development of viral vector systems for delivery into the central nervous system (CNS).

Due to the natural ability of viruses to efficiently transduce cells and tissues with foreign nucleic acid, they have attracted attention as a means of gene delivery since the 1980s (Friedmann, 1976). Viral vectors are engineered such that their wild type virus’ genome is replaced with a transgene of interest. Production of said vectors is normally accomplished by co-transfecting cells with multiple plasmids. One plasmid contains the desired transgene adjacent to the required packaging signals, and the other plasmids encode and thus provide all proteins necessary for vector formation in *trans*. As of 2018, over 3,000 gene therapy clinical trials have been initiated worldwide (with ~2% targeting neurodegenerative diseases; Ginn et al., 2018), and delivery *via* recombinant retro-, lenti-, or adeno-associated virus is employed in around 35% of these^1^. Simple recombinant retroviral vectors (based on γ-retroviruses) were used in the first gene therapy proof-of-principle study, aiming to correct a severe combined immunodeficiency disorder (SCID) in 1995 (Blaese et al., 1995). Tragically, the retroviral vector used in clinical trials induced severe T-cell leukemia in several children 2–5 years after gene therapy, and one of these children died. The insertion of the retroviral vector cassette in the proximity of a proto-oncogene, which then led to an uncontrollable expression of the gene, was determined to be the cause of leukemia, dramatically highlighting the limitations of γ-retroviral vector-based gene therapy (Kantor et al., 2014b). Furthermore, γ-retroviral vectors are not capable of transducing postmitotic cells, a huge disadvantage when targeting the CNS. Infection of slowly dividing cells is possible but is highly inefficient because these retroviral vectors rely on nuclear membrane disassembly for nuclear transportation (Miller et al., 1990; Lewis and Emerman, 1994). As such, simple retroviral vectors are not good candidates for gene therapy of neurodegenerative diseases.

## Lentiviral Vectors (LVS): Basic Biology

Unlike γ-retroviruses, lentiviruses [a different genera in the retroviridae family, exemplified by human immunodeficiency virus type-1 (HIV-1)] evolved a mechanism that exploits host-protein machinery to achieve efficient nuclear import through the intact nuclear membrane (Lewis and Emerman, 1994). Subsequently, these viruses have been engineered into useful viral vectors, as they are capable of transducing nondividing or terminally differentiated cells (e.g., postmitotic neurons) with high efficiency (reviewed in Kantor et al., 2014a). Since the first publication demonstrating the efficient transduction of lentiviral vectors into post-mitotic neurons *in vivo* (Naldini et al., 1996), thousands of studies have probed the use of HIV-based vectors for gene delivery into the CNS (Azzouz et al., 2002; Bayer et al., 2008; Kantor et al., 2011). HIV-based vectors have been demonstrated to transduce most cell types of the brain, including neuronal stem cells, neurons, astrocytes, and oligodendrocytes (Blömer et al., 1997; Consiglio et al., 2001; Azzouz et al., 2002; Jakobsson et al., 2003). Furthermore, HIV-based vectors are capable of sustaining long-lasting transgene expression in the brain (Bayer et al., 2008; Kantor et al., 2011). This last point is of the utmost importance, as continuous, long-lasting production of the therapeutic gene-of-interest (thus providing permanent steady-state “dosing” after a single administration of virus) is essential for gene therapy applications in the CNS.

**Figure 1:**
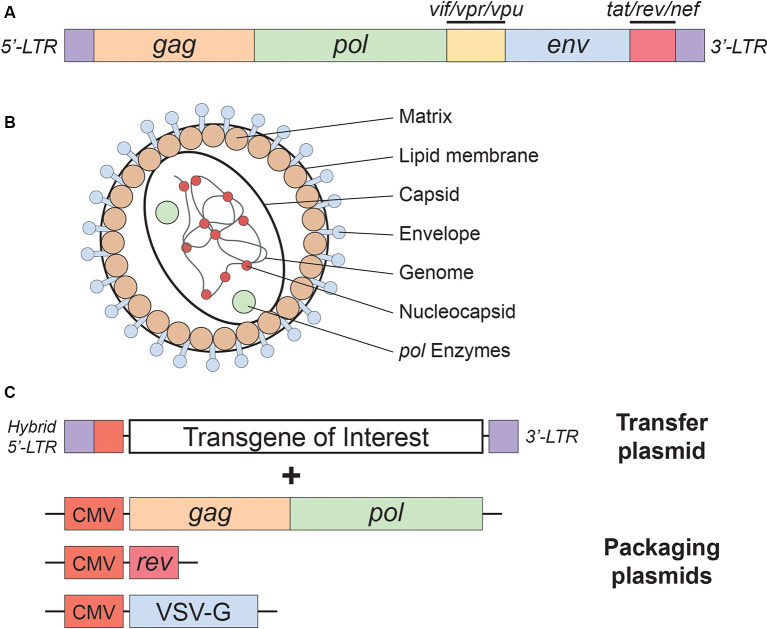
Lentivirus basics. **(A)** Simplified schematic of the wild-type human immunodeficiency virus type-1 (HIV-1) genome. **(B)** Lentivirus particle structure. **(C)** Plasmids used in the current (3rd generation) lentivirus packaging system. See the main text for a detailed description of the lentivirus packaging system; see Table 1 for lentivirus envelope proteins.

As mentioned above, lentiviral vectors (LVs) are derived from the HIV-1. The lentiviral genome occupies ~10.7 kbs of positive-sense single-stranded RNA (Figure 1A), of which two copies are packaged inside a lipid-enriched viral shell that is ~100 nm in diameter (Figure 1B). In recombinant LVs (which lack all the HIV-1 ORFs but retain several critical non-coding elements, detailed below), this results in a packaging capacity of approximately 10 kb. The genome encodes structural and enzymatic genes including *gag* and *pol*, respectively. The *gag* (group-specific antigen) encodes the viral matrix (MA), capsid (CA), and nucleoproteins (NC). The enzymatic machinery of the virus consists of reverse transcriptase (RT), protease (PR), and integrase (IN). The virus uses its envelope for attachment and entry into the host cell. Construction of heterologous envelope proteins for pseudotyping viral particles was one of the major steps in dramatically diversifying the tropism of lentiviral vectors. Furthermore, it greatly enhanced the safety profile of the vector (reviewed in Kantor et al., 2014a). Lentiviral vectors can be pseudotyped with a wide variety of envelope proteins; many of them, including Mokola virus (MV), Ross River virus (RRV) and Rabies virus (RV) shown strong neurotropic tropism (Table 1; also reviewed in Cronin et al., 2005). However, the most commonly employed envelope is vesicular stomatitis virus protein G (VSV-G), characterized by its extremely broad cellular tropism.

**Table 1:** Envelope proteins used for pseudotyping lentiviral vectors (LVs).

Envelope	Hosts	CNS Tropism
VSV-G	Mouse, rat, pig, dog, human	Non-selective
Mokola virus	Mouse, rat	Non-selective
Rabies virus	Mouse, rat	Prefers neurons; efficient axonal transport
LCMV	Mouse, rat	Prefers astrocytes; some expression in neurons
RRV	Mouse, rat, human cells *(in vitro)*	Non-selective

*VSV-G, vesicular stomatitis virus G-protein; LCMV, lymphocytic choriomeningitis virus; RRV, Ross River Virus. Adapted from Kantor et al. (2014a)*.

Following the entry into host cells *via* receptor binding and fusion of the viral envelope with the cell membrane, reverse transcription (RT) reaction takes place in the cytoplasm (see Figure 3). The RT enzyme mediates a complex reverse transcription process which results in the generation of double-stranded (ds), linear, DNA. For this reaction to take place, the LV genome must include a primer binding site (PBS) and a polypurine tract (PPT). The PBS is responsible for RT initiation, as a tRNA^Lys3^ binds to it and is used as a primer, and it is also critical in the second template exchange that occurs. The PPT contains a purine-rich stretch that survives RNase H—mediated degradation of the positive-stranded RNA, and thus acts as a primer for RT to create positive-stranded DNA (reviewed in Kantor et al., 2014a). The viral DNA corresponds with its genomic RNA but contains a duplicate of the U3 and U5 regions at the 5′LTR (long terminal repeat) and 3′LTR, respectively. The U3 region harbors the promoter sequence, while the U5 region carries the poly-A signal (reviewed in Kantor et al., 2014a). The linear dsDNA is then imported into the nucleus and serves as a precursor for integration. Integrase (IN) protein mediates this process by catalyzing binding and cleaving within the *att* sites located on both ends of the DNA (Colicelli and Goff, 1985; Craigie et al., 1990; Leavitt et al., 1992). Following integration, the viral DNA acts as a part of the host’s DNA and is therefore replicated along with it, and passed on to the cell’s progeny (Buchow et al., 1989). The RT and PR proteins are essential for LV production; contrarily, the vector can sustain its life-cycle without IN. Unsurprisingly, this fact has been exploited and lead to the formation of integrase-deficient lentiviral vectors (IDLVs), which provide some significant advantages over the conventional integrase-competent lentiviral vectors (ICLVs), a topic that will be discussed later in this review.

**Figure 2:**
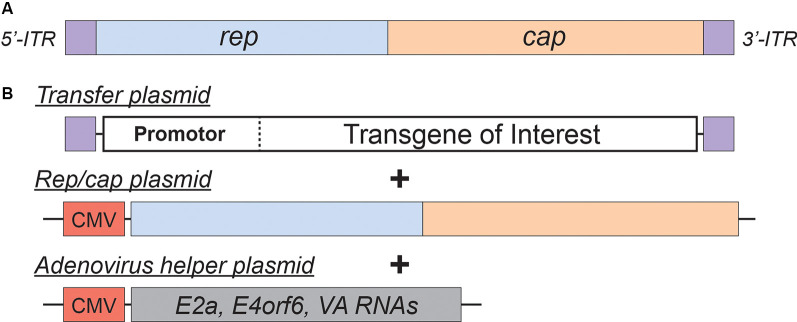
Adeno-Associated Virus (AAV)basics. **(A)** Simplified schematic of the wild-type AAV genome. **(B)** Plasmids used in the current AAV packaging system. See the main text for a detailed description of the AAV packaging system; see Table 2 for a comparison of common AAV serotypes.

**Figure 3:**
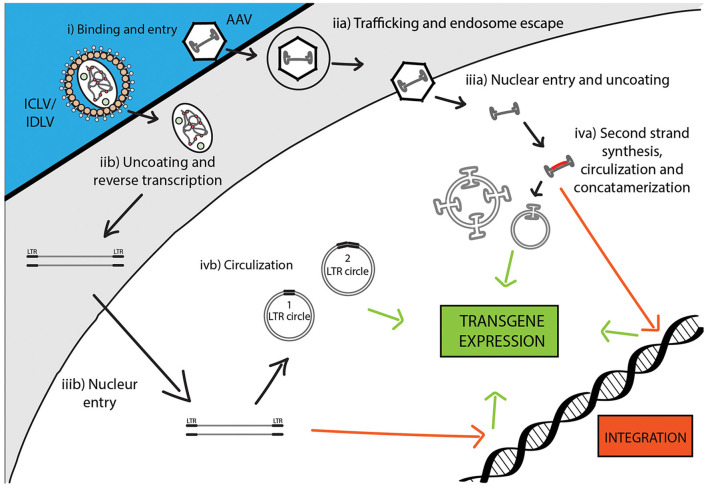
Comparison of recombinant Integrase-Competent Lentivirus (ICLV), Integrase-Deficient Lentivirus (IDLV), and AAV life cycles. ICLV, IDLV, and AAV bind and enter target cells (i). AAV particles escape from endosomes into the cytoplasm (iia), then enter the nucleus and un-coat (iiia). After un-coating, a host polymerase performs second strand synthesis, leading to circularization and concatemerization of the AAV vector; a small percent integrates randomly into the host genome. Transcription occurs from all forms of the AAV transgene (iva). Uncoating and reverse transcription of ICLVs and IDLVs occur in the cytoplasm (iib). The dsDNA product is then imported into the nucleus (iiib). Some of this DNA integrates into the host genome, while the majority recombines into one- or two-LTR circles and remains episomal (ivb). Transcription occurs from all forms of the transgene, but rates of integration and circle formation differ between IDLV and ICLV (see Table 3).

In addition to the core proteins, *gag* and *pol*, lentiviruses such as HIV-1 harbor six additional genes: two regulatory (*rev* and *tat*), and four accessory genes (*nef*, *vif*, *vpr*, and *vpu*), involved in the viral entry, replication, and particle release (Coffin et al., 1997). The accessory products can be deleted from the packaging cassette as they are not necessary for LV production. Their exclusion not only enhances the safety of the vector but also creates a space for the insertion of transgenic sequences (Naldini et al., 1996; Blömer et al., 1997; Kafri et al., 1997; Dull et al., 1998). This realization led to the construction of second-generation packaging cassettes that harbor only the *tat* and the *rev* genes (Zufferey et al., 1997). The *tat* gene encodes a trans-activator of transcription *(Tat)* protein responsible for enhancing HIV-1 expression. The replacement of the endogenous HIV-1 promoter in the U3 region of the 5′LTR with a strong promoter, such as Rous sarcoma virus (RSV) or cytomegalovirus (CMV), creates independence of the virus from *tat*. Still, some of the second-generation packaging plasmids continue to harbor *tat*, as it seems to have a positive effect on the viral production titer. However, *tat* is excluded in third-generation packaging systems (Figure 1C), which are also characterized by the separation of the *gag/pol* and *rev* sequences into two different cassettes, and are the safest LVs to date (Dull et al., 1998). In contrast to *tat*, the *rev* gene is indispensable, as its protein product is responsible for exporting full-length and partially spliced RNAs from the nucleus to the cytoplasm (Cockrell et al., 2006). Another improvement present in the current (third-generation) packaging systems is the replacement of the virus’ weak polyadenylation signal (poly-A) for either SV40 or bovine/human growth hormone (bGH/hGH), which potentiate mRNA stability (Dull et al., 1998; Cockrell et al., 2006). Also, the incorporation of a woodchuck hepatitis virus posttranscriptional regulatory element (WPRE) and a central polypurine tract (cPPT) into the expression cassette further up-regulated RNA stability, transcription efficiency, and viral titer (Zufferey et al., 1999; Zennou et al., 2000). Importantly, the above modifications neither reduced vector yield nor hampered the ability of LVs to transduce nondividing cells, such as terminally differentiated neurons (Dull et al., 1998; Zufferey et al., 1999; Zennou et al., 2000; Cockrell et al., 2006). Together, they significantly reduced the likelihood of generating recombination-competent retroviruses (RCR), thus contributing to the vector’s superb safety.

### Non-integrating Lentiviral Vectors

Despite the aforementioned advances in vector safety, employment of retroviral vectors in clinical trials is hampered by a relatively high risk of insertional mutagenesis (reviewed in Kantor et al., 2014a,b). It is important to note that the likelihood of insertional mutagenesis is considered to be lower in lentiviral vectors compared to their γ-retroviral vector counterparts. For example, in the tumor-susceptible mouse model, transplantation of γ-retroviral vector-transduced hematopoietic cells resulted in an accelerated tumorigenic process, whereas no additional adverse events were detected with lentiviral vectors (Montini et al., 2006). Moreover, it has been shown that a higher quantity of lentiviral vectors is necessary to cause an oncogenic risk similar to that of γ-retroviral vectors (Montini et al., 2009). Nevertheless, lentiviral vectors are not completely detached from this problem. An Equine infectious anemia virus-derived vector has been reported to be associated with the formation of tumors in the livers of mice following *in utero* and neonatal vector administration (Themis et al., 2005).

To avert insertional mutagenesis, integrase-deficient lentiviral vectors (IDLVs) have been developed (see Figure 3). The IDLVs can be generated by introducing non-pleiotropic mutations within the open reading frame (ORF) of the *int* ORF (Engelman et al., 1995). Such mutations have been shown to specifically target the integration process without significantly affecting other steps of the LV life cycle (Figure 3 and Table 3). We previously reported that IDLV genomes are indeed capable of being expressed *in vitro* and *in vivo*, however, they do demonstrate lower expression levels compared to ICLVs (Bayer et al., 2008; Kantor et al., 2009). Still, these reduced expression levels are often sufficient for correcting genetic disorders in animals (Philippe et al., 2006; Yáñez-Muñoz et al., 2006). We demonstrated that the reduced level of IDLV expression is attributed to the formation of a repressive chromatin structure around the episomal DNA (Kantor et al., 2009). Furthermore, we showed that the reduced expression of IDLVs can be corrected by removing repressive factors such as histone deacetylases (HDACs) either *via* *in-cis* or *in-trans* methods. For example, we demonstrated that the deletion of negative transcription elements (NTE) located within U3-region of the 3′LTR resulted in significant activation of IDLV expression in both *in vitro* and *in vivo* experiments (Philippe et al., 2006; Yáñez-Muñoz et al., 2006; Kantor et al., 2011). More recently, we showed that the addition of the transcriptional enhancers, such as Sp1 within the viral expression cassette can further stimulate packaging efficiency and transgene expression *in vitro* and *in vivo* (Ortinski et al., 2017). Here, we carefully analyzed the levels and duration of transgene expression, the integration rate, and the overall therapeutic potential of IDLV vectors in comparison to their integrase-competent counterparts (Bayer et al., 2008; Kantor et al., 2011; Saida et al., 2014). Importantly, IDLV-mediated proviral integration into host’s cell chromosomes occurred in approximately 1/3850 HeLa cells and approximately 1/111 mouse cerebellar neurons *in vivo* (Bayer et al., 2008; Kantor et al., 2011); that is ~500-fold lower than the integration rate of ICLV. To examine the therapeutic potential, IDLVs and ICLVs carrying therapeutic cargo encoding an enhancer of the ubiquitin-proteasome pathway were injected into the cerebellum of spinocerebellar ataxia type 3 model mice (SCA3 mice). Remarkably, IDLV-injected SCA3 mice showed significantly improved rotarod performance even 1-year post-injection (Saida et al., 2014). Furthermore, immunohistochemistry at 1-year post-injection showed a dramatic reduction of mutant aggregates in Purkinje cells of both IDLV- and ICLV-injected SCA3 mice. Many other laboratories have also demonstrated efficient use of IDLVs for the transduction of most cell types in the brain (Saida et al., 2014; Lu-Nguyen et al., 2016; Ortinski et al., 2017). More recently, we established and optimized IDLV vectors as a means for safe and efficient delivery of CRISPR/Cas9 components (Ortinski et al., 2017; Vijayraghavan and Kantor, 2017). Importantly, we reported that IDLV vectors are capable of attaining a strong and sustained CRISPR/Cas9 expression in dissociated post-mitotic neurons and in the rat brain in post-mitotic neurons *in vitro* and *in vivo*. Furthermore, we demonstrated that IDLV-CRISPR/Cas9 vectors are significantly less prone to induce off-target DNA perturbations, and as such are more specific and safe comparing with their integrase-competent counterparts (Ortinski et al., 2017). These studies altogether suggest that IDLVs may provide an effective and safe means of delivery of therapeutic transgenes into the CNS.

**Table 2:** Common Adeno-Associated Virus (AAV) serotypes.

		Tissue Tropism	
Serotype	Origin	*Mouse*	*Primate*
AAV1	Human	CNS, retina, liver, heart, muscle, airway, pancreas	CNS, muscle
AAV2	Human	CNS, retina, liver, muscle, kidney	CNS, retina
AAV3	Human	Muscle	Liver
AAV4	Non-human primate	CNS, retina, lung, kidney	Lung
AAV5	Human	CNS, retina, muscle, airway	-
AAV6	Human	Heart, muscle, airway	Airway
AAV7	Rhesus macaque	CNS, retina, liver, muscle	-
AAV8	Rhesus macaque	CNS, retina, liver, heart, muscle, pancreas, kidney	CNS, liver
AAV9	Human	CNS, retina, liver, heart, muscle, lung, pancreas, kidney, testes	CNS, retina, heart
AAVrh10	Rhesus macaque	CNS, retina, liver, heart, muscle, lung, pancreas, kidney	-
		
AAV-AS	Derived from AAV9	CNS—transduction improved 6–15x vs. AAV9	
AAV-BR1	Derived from AAV2	Brain endothelium	
Olig001	Derived from AAVs1, 2, 6, 8, and 9	Oligodendrocytes	
TM6	Derived from AAV6	Microglia	
AAV-DJ	Derived from AAVs 2, 4, 5, 8, and 9	Liver	
AAV-DJ/8	Derived from AAV-DJ	Liver, CNS	
rAAV2retro	Derived from AAV2	CNS—efficient retrograde transduction	
PHP.B	Derived from AAV9	CNS—transduction improved ~40x vs. AAV9	
PHP.S	Derived from PHP.B	Peripheral nervous system	
PHP.eB	Derived from PHP.B	CNS—transduction rate further improved vs. PHP.B	

*Adapted from Grimm et al. (2008); Lisowski et al. (2015); Choudhury et al. (2016b); Deverman et al. (2016); Körbelin et al. (2016); Powell et al. (2016); Rosario et al. (2016); Tervo et al. (2016) and Chan et al. (2017)*.

**Table 3:** Viral vector comparison.

	ICLV	IDLV	AAV
Insert size	10 kb	10 kb	4.7 kb
Integration rate*	~30%	~0.05%	Up to 1%
Risk of insertional mutagenesis	Medium	Low	Low
Cytotoxicity	Low	Low	Low
Pre-existing Ab	Low	Low	High
Immunogenicity	Low	Low	Medium
Neurotropism**	High	High	High
Titer	Medium	Medium	High

*in vitro. **With optimized pseudotype/serotype. ICLV, Integration-competent lentivirus; IDLV, Integration-deficient lentivirus; AAV, Adeno-associated virus. Adapted from McCarty et al. (2004) and Kantor et al. (2011).

## Adeno-Associated Viral Vectors (AAV Vectors): Basic Biology

Adeno-associated viral vectors are the most frequently utilized platforms for the delivery of therapeutic genes (reviewed in Kantor et al., 2014a). These recombinant AAV (rAAV) vectors were engineered from the wild type virus, which belongs to the *Dependovirus* genus of the Parvoviridae family. As indicated in the genus name, the virus depends on coinfection of another virus (adenovirus or HSV) for replication in host cells (reviewed in Lentz et al., 2012). The packaging-competent form of the AAV genome is represented by a 4.7 kb ssDNA (Figure 2A). The genome itself appears quite simple: two ORFs, *rep* and *cap*, flanked by a pair of 145 bp inverted terminal repeats (ITRs; Lusby et al., 1980; Srivastava et al., 1983; Sonntag et al., 2010). However, the wild-type AAV genome encodes eight proteins in total. The *rep* ORF encodes four isoforms of the Rep protein (each combination of two promotors and two splice variants). The long isoforms (Rep78/68; named for their molecular weight) are responsible for replication and integration of the viral genome, and the short Rep52/40 isoforms mediate genome packaging. The *cap* ORF encodes the structural capsid proteins VP1, VP2, and VP3. Through a combination of transcriptional and translational mechanisms beyond the scope of this review, VP1/VP2/VP3 are produced at a ratio of about 1:1:10, respectively (Kronenberg et al., 2001); Sixty copies of VP1/2/3 in the same ratio make up each icosahedral AAV particle. Lastly, assembly activating protein (AAP) is encoded by a cryptic, out-of-frame ORF contained within *cap*; AAP is involved in trafficking capsid proteins to the nucleolus (the site of virion assembly) and is also instrumental in the capsid assembly process (reviewed in Smith, 2008).

During infection, AAV enters cells through receptor-mediated endocytosis, which occurs *via* clathrin-coated pits (Bartlett et al., 2000). As AAV encodes no envelope protein, the viral capsid determines the tissue specificity or tropism. Once inside the cell, the virus escapes from the early endosome and translocates into the host’s nucleus where virion uncoating is completed (Figure 3). The hairpin endings of the ssDNA genome are then recognized by a host DNA polymerase and are subsequently filled in to create dsDNA (Ferrari et al., 1996). At this stage, WT AAV is capable to efficiently and site-specifically integrate (onto chromosome 19 in humans) into the host cell genome (Deyle and Russell, 2009). The integrated form can be released from the host’s genome following coinfection with a helper virus (Adenovirus or HSV-1) or cellular stress, which leads to a lytic cycle where AAV transcription and DNA replication are reactivated to produce AAV viral particles (Kotin et al., 1990; Samulski et al., 1991). In the absence of a helper virus, wild-type AAV DNA can also be retained in the nucleus in linear and circular episomal forms (Duan et al., 1998; Schnepp et al., 2005).

AAV is an ideal virus to modify into a delivery vector for several reasons. Most importantly, the virus has no known associated pathologies and causes a mild immune response in humans. Second, the AAV genome can be preserved for extended periods in episomal forms, and thus presents an opportunity for prolonged transgene expression. Furthermore, AAVs are common in nature, and as such many serotypes exist, with varied tropisms (Table 2). Lastly, the AAV genome is well-understood, so the consequences of genetic manipulations can reasonably be predicted. For these reasons, over the last 30 years, a substantial effort has been devoted to transforming AAV into one of the gold-standard platforms for gene therapy. In this time, several major milestones have been achieved towards creating a safe and efficient rAAV toolkit. First, it was found that the stem-loop-forming inverted terminal repeats (ITRs) are the only cis-acting elements required for both genome replication and packaging of the genome into virions (Lusby et al., 1980; Nash et al., 2008). Unsurprisingly, this led to the creation of a packaging plasmid which provides the *rep* and *cap* genes *in trans*. Thus, in recombinant AAVs, nearly the entire genome is replaced with a transgene of interest, yielding a functional packaging capacity quite close to the 4.7 kb WT genome size. Furthermore, the split of these genes from the vector plasmid is critical to prevent the formation of WT AAV during rAAV production (reviewed in Kantor et al., 2014a). As the necessary rep gene is no longer packaged, this separation of the viral cassette also causes rAAV to lose the site-specificity of its integration into human chromosome 19. Instead, rAAVs appear to integrate randomly at a low rate (integration occurs in 0.1–1% of cells), with the vast majority of DNA being maintained as episomes (reviewed in Kantor et al., 2014a). Second, the helper function needed for AAV replication and viral production was initially provided by co-infecting the production cells with Adenovirus or HSV-1. However, this method results in the contamination of rAAV preparations with Adenovirus or HSV particles. To solve this problem, researchers constructed a separate cassette carrying only the essential adenovirus helper genes: E1a, E1b, E2a, E4orf6, and viral-associated RNA genes (Xiao et al., 1998). Importantly, HEK293T cells, which are commonly used for rAAV production, already express E1a and E1b; as such, these genes have been excluded from the helper cassette (Xiao et al., 1998). The optimized rAAV production protocol (Figure 2B) thus utilizes three plasmids transiently transfected into HEK293T producer cells: the vector plasmid with the transgene-of-interest flanked by AAV ITRs, the packaging plasmid containing the *rep* and *cap* genes from a specific AAV serotype, and the adenovirus helper plasmid (Xiao et al., 1998). These revolutionary advancements have enabled large-scale production of pure rAAV with low immunogenicity, which can be used for a variety of gene transfer applications, including human gene therapy.

More recently, researchers have developed second-generation rAAV vectors with modified capsids that enhance tissue selectivity as well as evading neutralizing host antibodies. An understanding of the biology of naturally occurring serotypes allowed scientists to create hybrids and then engineer these new vector capsids. AAVs use specific regions of their capsid proteins to bind to receptors on the host’s cellular membrane; a virus’s serotype is determined by the particular amino acid residues that make up these hypervariable loop regions. These variations affect which receptors the capsid proteins bind to, and thus different serotypes confer different tropisms. Furthermore, it has been demonstrated that serotype plays an essential role in viral trafficking from the host’s cell membrane to the nucleus as well as in the virion uncoating process, which may in turn control the efficiency of transduction and expression (Keiser et al., 2011).

Over 100 AAV serotypes and variants have been described so far, with the most studied and utilized being AAV2 (Summerford and Samulski, 1998; Summerford et al., 1999; Gao et al., 2005; Wu et al., 2006), and reviewed in Mitchell et al. (2010). However, researchers have also contributed significantly to this remarkable variety by creating pseudotyped viral variants. AAV pseudotypes are usually created by altering the packaging plasmid such that it carries *cap* from the serotype-of-interest along with *rep* from AAV2 while keeping AAV2 ITRs in the transgene-carrying plasmid. The resulting viruses are denoted using a slash: for example, AAV2/5 indicates a virus containing the genome of serotype 2 packaged in the capsid from serotype 5 (reviewed in Mitchell et al., 2010). AAV2/5 in particular demonstrates improved affinity for neuronal cells that are not efficiently targeted by AAV2/2 and is distributed more widely in the brain, allowing for greater transduction efficiency (see below). Another method to expand AAV tropism is to create hybrid capsids derived from multiple serotypes (reviewed in Castle et al., 2016). Multiple groups have further engineered these second-generation AAV vectors using both rational design-based and directed evolution-based approaches (reviewed in Gray et al., 2010). Together, these newly engineered AAV vectors offer a broad range of tropisms to meet a variety of experimental and therapeutic needs.

Due to the advances described above, AAV is the platform of choice for viral gene delivery into the CNS (Tables s 2, 3; also reviewed in Gray et al., 2013). The following serotypes have been effectively used in the CNS: AAV2/1, AAV2/5, AAV2/6, AAV2/8, AAV2/9, and the recently engineered PhP.eB (Chan et al., 2017). When injected into the brain, AAV2/1 and AAV2/5 are more efficient than AAV2/2 at transducing both neurons and glial cells, in multiple brain regions of rats and nonhuman primates (Burger et al., 2004; Mandel and Burger, 2004). In contrast AAV2/7, AAV2/8, and AAV2/9 primarily transduce neuronal cells, with AAV2/9 exhibiting the widest spread from the site of injection (Cearley and Wolfe, 2006). Axonal transport varies amongst the AAV serotypes and can be exploited to infect both the directly-targeted cell types as well as the projection field of those cells. For example, when injected into the ventral tegmental area, AAV2/1 and AAV2/9 have shown a high level of spread in both directions along with axonal projections (Cearley and Wolfe, 2006). One of the challenges of targeting the brain is identifying vectors that can cross the blood-brain barrier (BBB) so that, ideally, gene therapy can be administered peripherally. To this end, Foust et al. (2008) and Duque et al. (2009) demonstrated that AAV2/9 administered intravenously crosses the BBB of mice and cats, in both neonatal and adult animals; similarly, Gray et al. (2013) showed that AAV2/8 was able to cross the BBB in mice, although to a lesser extent than AAV2/9 (Hester et al., 2009). Importantly, both neurons and astrocytes were transduced by intravenously injected AAV2/9 vectors, demonstrating that it is possible to deliver gene therapy to a large portion of the brain and spinal cord without having to inject directly into the CNS (Foust et al., 2008; Duque et al., 2009).

In addition to the options provided by simple AAV pseudotyping, a growing array of engineered AAV serotypes are now available, which display a range of useful properties (Table 2). These include Olig001 and TM6, which selectively transduce oligodendrocytes and microglia, respectively, when delivered to the CNS. As glial cells are known to play important roles in the neurodegenerative process, the ability to target glia selectively may prove critical for future therapeutic applications. Also notable is rAAV2-retro, a derivative of the AAV2 capsid (*via* directed evolution) which displays robust retrograde transduction across synapses. This is a particularly valuable tool for basic research into brain connectivity. Furthermore, selective delivery to sets of neurons defined by their downstream connectivity may prove to have therapeutic applications. Lastly, the recently engineered PHP.eB serotype consistently exhibits efficient transduction of the CNS *via* systemic delivery in adult animals (Chan et al., 2017). Furthermore, in *in vivo* studies, it has consistently shown higher transduction rates comparing to those of AAV2/9. Indeed, the intravenously injected (IV) PHP.eB-AAV found to be superior to AAV2/9 in both the expression level per cell and the number of transduced cells; its transduction has been reported to be close to 100% in neurons in the cortex and striatum, and over 75% in cerebellar Purkinje cells (Chan et al., 2017). Notwithstanding the enhanced CNS tropism in mice, AAV-PHPeB failed to efficiently transduce the CNS in nonhuman primates following intravenous infusion. Further investigation will be required to determine if the efficient transduction of AAV-PHPeB extends beyond the model in which it was originally tested (Hordeaux et al., 2018). Furthermore, the extent of pre-existing immunity towards this serotype shall be determined; as the presence of anti-AAV2/9 vector neutralizing antibodies (closely relating to PHPeB) in the human population presents a significant challenge for any AAV2/9-based gene therapy. One strategy for circumventing this potential problem would be to use alternate routes of administration. For example, delivery into CSF *via* intrathecal injection has been tested as an alternative route to IV injections (Federici et al., 2012; Samaranch et al., 2012; Gray et al., 2013). Although more invasive than an IV injection, intra-CSF administration has proven much more efficient for targeting cells in the spinal cord. Consistently, many groups have now demonstrated that intra-CSF delivery of AAV2/9 results in widespread transgene expression in large experimental animals (Haurigot and Bosch, 2013). Remarkably, it has been demonstrated that AAV-mediated transgene expression in the brain is long-lasting: more than a year in mouse (Klein et al., 1999), at least 6 years in primates (Rivera et al., 2005), and over 8 years in dogs (Niemeyer et al., 2009). Most importantly, a therapeutic level of expression has been detected 8 years post-transduction in the human brain (Leone et al., 2012). Significantly, clinical-grade AAV vectors have been routinely manufactured at the high titers for CNS delivery using human-suitable protocols. Furthermore, AAV-based treatments for CNS disorders are as of this moment finding their first success in the clinic: Zolgensma, an AAV9-based gene replacement therapy for spinal muscular atrophy was approved by the FDA in 2019. A detailed description of how AAV vectors have been developed into a CNS gene-transfer products can be found in (Kantor et al., 2014a).

## Overview of CRISPR/Cas9-Based Gene-Editing Systems

The CRISPR and CRISPR-associated protein (Cas) system has recently emerged as a revolutionary genetic tool for genome- and epigenome- editing in the CNS. CRISPR/Cas has already advanced our understanding of complex neurologic diseases by enabling the rapid generation of novel, disease-relevant animal models. Furthermore, as will be discussed comprehensively in this review, CRISPR/Cas-based editing provides us with an unprecedented tool to treat neurodegenerative diseases (NDDs). Here, we will review the development and use of CRISPR-mediated genome engineering.

The CRISPR/Cas system offers notable advantages over earlier genome-editing technologies, the two most prevalent of which are zinc finger nucleases (ZFNs) and transcription activator-like effector nucleases (TALENs). ZFNs are relatively small, and once successfully designed can be highly effective, but targeting a ZFN construct to a specific DNA sequence is a non-trivial, time-consuming process. Targeting of TALENs, meanwhile, is relatively straightforward compared to ZFNs (though not as simple as CRISPR), but the size of an active TALEN construct (a two-protein heterodimer totaling ~6 kb of coding sequence) often proves extremely challenging for delivery (van Haasteren et al., 2020). Nevertheless, while CRISPR has largely supplanted these technologies, its rapid development (particularly on the delivery front) was undoubtedly aided by previous work using ZFNs and TALENs. In particular, IDLVs have been employed to both map ZFN cleavage sites and deliver ZFN constructs *in vivo* (Yin et al., 2017).

In nature, CRISPR/Cas is a prokaryotic acquired-immunity mechanism that evolved to target and destroy the nucleic acid of phages, viruses, archaea, and other invading organisms (Barrangou et al., 2007; Sorek et al., 2008). The CRISPR/Cas system encompasses a variety of components that differ widely in the mechanism of action (reviewed in Makarova and Koonin, 2015; Makarova et al., 2015). The overall diversity of the system is tremendous, consisting of six Cas enzyme types (I–VI), and at least 29 subtypes (Koonin et al., 2017). Despite the complexity of the Cas family, all systems share CRISPR RNA [guide RNA (gRNA) and trans-activating RNA (tracrRNA)]-defined targeting specificity (Deltcheva et al., 2011; Jinek et al., 2012). The most attractive platform for gene-editing applications in humans derives from the class II CRISPR-associated enzyme Cas9, which acts as a single effector protein; in contrast, the class I Cas enzymes operate as multi-subunit protein complexes (reviewed in Shmakov et al., 2017). Herein, only Cas9-based systems will be discussed.

For gene editing applications, the two CRISPR RNAs mentioned above are combined into one small guide RNA (sgRNA), which greatly simplifies delivery. Cas9 itself can only bind to DNA at a specific sequence, known as its protospacer-adjacent motif (PAM). After PAM binding, the double-stranded DNA unwinds, allowing the Cas9-associated sgRNA to hybridize with the exposed DNA strand (the protospacer), assuming they are complimentary. If so, the catalytic domains of Cas9 then cleave both strands of the target DNA. Cas9’s unprecedented specificity has been rapidly exploited by scientists to fit a great range of applications, from basic science to translational research and medicine (Hsu et al., 2014). In turn, this early progress has inspired further efforts to develop novel CRISPR/Cas systems and apply them for a range of diseases, including NDDs.

One constraint of Cas9 is its dependency on the aforementioned PAM sequence to bind DNA. For example, the canonical PAM associated with the Cas9 nuclease of Streptococcus pyogenes (SpCas9) is the sequence 5′-NGG-3′ (Anders et al., 2014). Many other Cas9 proteins have been (and continue to be) isolated from other prokaryotes in nature which have different PAMs (Table 4). However, the efficiency of these Cas9 proteins varies, and to our knowledge, none have surpassed SpCas9. Thus, to increase coverage of potential target sites, rational engineering and evolution-based approaches have been employed to create new Cas9 variants with altered PAM specificities (Table 4). For example, Kleinstiver et al. (2015) used a series of positive selection screens in bacteria to identify mutants of SpCas9. They evolved three variants (VQR, EQR, and VRER) that recognize the novel PAM sequences NGAN/NGNG, NGAG, and NGCG, respectively. Another example is the Cas9 of Francisella novicida, which has been engineered to recognize a non-canonical 5′-YG-3′ PAM (Hirano et al., 2016). Recently, however, a more groundbreaking solution to the PAM specificity problem was reported, again from the Kleinstiver lab. Through a multi-step process of rational design, two significant SpCas variants were engineered: SpG, which is capable of targeting an expanded set of NGN PAMs, and a near-PAMless variant called SpRY (Walton et al., 2020). Collectively, SpG and SpRY enable unconstrained targeting using CRISPR-Cas9 nucleases across nearly the entire genome, with single base-pair precision. Using SpRY, the authors were able to correct mutations associated with human diseases located in previously “un-editable” regions of the genome (Walton et al., 2020).

**Table 4:** Major CRISPR-associated protein 9 (Cas9) isoforms.

Name	Construct size	sgRNA length	PAM sequence*
Naturally occurring		
SpCas9	4.1 kB	20 bp	NGG
StCas9	3.4 kB	20 bp	NNAGAAW
NmCas9	3.2 kB	24 bp	NNNNGATT
SaCas9	3.2 kB	21 bp	NNGRRT
CjCas9	2.9 kB	22 bp	NNNNRYAC
Engineered from SpCas9		
SpCas9	~4.1 kB	20 bp	NGG**
SpCas9-HF1	~4.1 kB	20 bp	NGG**
HypaCas9	~4.1 kB	20 bp	NGG**
evoCas9	~4.1 kB	20 bp	NGG**
VQR	~4.1 kB	20 bp	NGAN or NGNG
EQR	~4.1 kB	20 bp	NGAG
VRER	~4.1 kB	20 bp	NGCG
Cas9-NG	~4.1 kB	20 bp	NGN
xCas9	~4.1 kB	20 bp	NGN or GAW
SpG	~4.1 kB	20 bp	NGN
SpRY	~4.1 kB	20 bp	NRN > NYN

*Nucleotide codes: N—any base; W—A or T; R—A or G; Y—C or T. **Fidelity improved vs. WT SpCas9. Table adapted from Kleinstiver et al. (2015); Komor et al. (2018); Slaymaker et al. (2016); Chen et al. (2017); Adli (2018); Casini et al. (2018); Hu et al. (2018); Nishimasu et al. (2018) and Walton et al. (2020).

Another impetus for engineering Cas9 is to increase targeting specificity and minimize off-target effects (Mueller et al., 2018). Several studies have described Cas9 variants evolved to reduce off-target cleavages (Kleinstiver et al., 2016; Slaymaker et al., 2016; Chen et al., 2017; Kulcsár et al., 2017). Alternatively, an improvement in on-target CRISPR/Cas specificity can be achieved by modifying the secondary structure of the gRNA spacer region in such a way that it increases the thermodynamic barrier to gRNA binding at off-target sites (Kocak et al., 2019).

**Figure 4:**
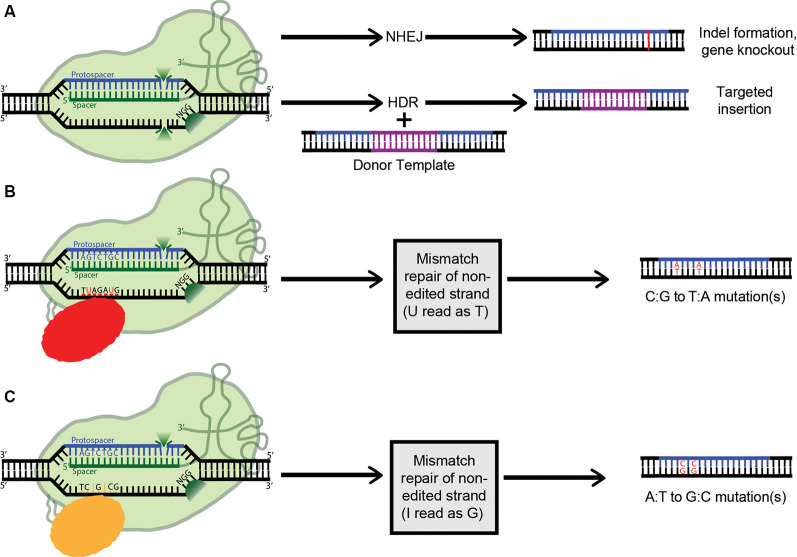
Applications of clustered regularly interspaced short palindromic repeats (CRISPR) technology** (A)** Active Cas9 introduces a double-stranded DNA break, which is repaired *via* non-homologous end joining (NHEJ), creating indels. Alternatively, if a dsDNA donor template is provided, the dsDNA break can be repaired by homologous recombination, resulting in a targeted insertion. **(B)** Cytosine Base Editors catalyze the conversion of all cytosines within a 5–6 nucleotide window to uracils. Uracil is then read as thymine during replication, completing the C:G to T:A conversion. **(C)** Similarly, Adenosine Base Editors (ABEs) catalyze the conversion of all adenosines within a 5–6 nucleotide window to inosines. Inosine is then read as guanine during replication, completing the A:T to G:C conversion.

As mentioned above, when co-expressed with CRISPR RNA, active Cas9 endonuclease cuts both strands of the target DNA, introducing a double-stranded break (DSB). Eukaryotes predominantly repair DSBs *via* the error-prone non-homologous end joining (NHEJ) pathway, which leads to the formation of small insertions or deletions (indels) in the target sequences (Figure 4A). Alternatively, if a repair template is supplied with homology to the target site, the host’s repair machinery activates homology-directed repair (HDR), resulting in error-free replacement of the target DNA (Figure 4A). However, HDR is typically characterized by lower efficiency than NHEJ-mediated repair. Furthermore, as it is not active in post-mitotic cells, HDR has a very limited ability to introduce such specific changes in the brain. Also, the DSBs needed to trigger efficient HDR increase the possibility of off-target effects, and even on-target HDR can have negative effects on cells (Haapaniemi et al., 2018; Ihry et al., 2018). This limitation motivated the development of single-base-pair editing and prime-editing technologies to enable precision genome editing in post-mitotic tissues such as the brain (discussed in detail below and reviewed in Komor et al., 2018; Anzalone et al., 2019).

**Figure 5:**
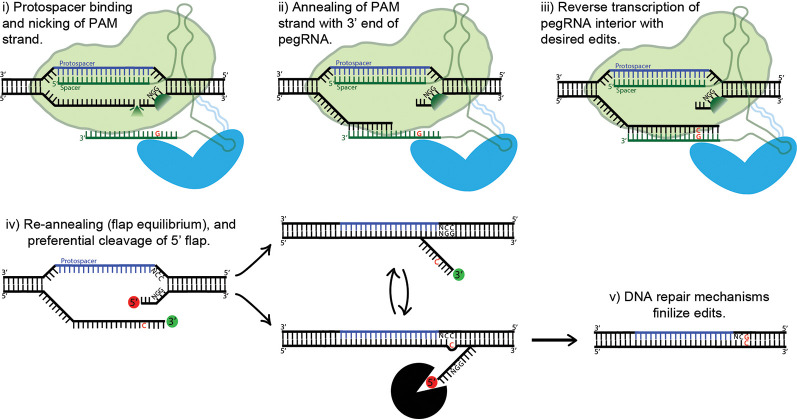
Proposed mechanism of prime editing. First, the 5’ end of the pegRNA binds to the protospacer of the target DNA and the protospacer-adjacent motif (PAM) strand is nicked (i). The nicked PAM strand then hybridizes with the primer binding site (PBS) at the far 3’ end of the pegRNA (ii). The interior of the pegRNA then serves as a template for reverse transcription, which extends from the free 3’-OH of the PAM strand (iii). The prime editing complex then disengages, leaving the target site with two redundant PAM strands, or “flaps” (iv). The unedited 5’ flap is preferentially degraded by cellular endonucleases, allowing the edited 3’ flap to hybridize with the non-PAM strand. Finally, DNA repair mechanisms transfer the desired edits to the non-PAM strand (v).

**Figure 6:**
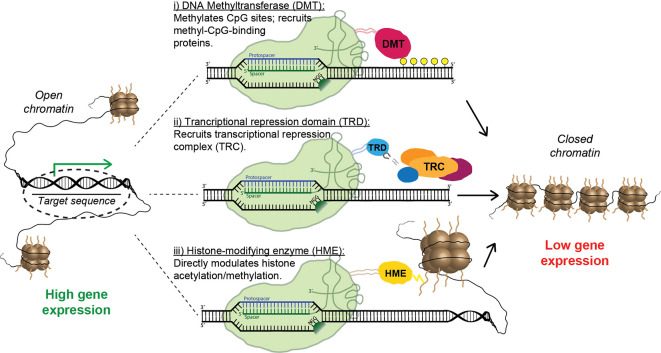
Strategies for epigenetic repression of risk-factor genes using Cas9 fusion proteins. Fusions containing the catalytic domain of a DNA methyltransferase cause targeted methylation of CpG sites and the recruitment of inhibitory methyl-CpG-binding proteins (i). Alternatively, a transcriptional repression domain (TRD) can be fused to Cas9, leading to the direct recruitment of transcriptional repression complexes (ii). Finally, multiple forms of inhibitory histone-modifying enzymes can be fused to Cas9, altering histone acetylation/methylation patterns and causing the formation of closed chromatin (iii).

Lastly, the ability of Cas9 to sequence-specifically bind DNA is of immense value in and of itself, independent of its catalytic activity. Indeed, for many theoretical applications, Cas9 endonuclease activity would be detrimental. To address this, mutations were identified in the RuvC (D10A) and HNH (H840A) nuclease domains which destroy the catalytic activity of Cas9 while maintaining its RNA-guided DNA-targeting capacity (Jinek et al., 2012; Qi et al., 2013). Cas9 is thus transformed from a targeted nuclease to a site-specific DNA recognition module. This exceptional modularity has motivated many groups to repurpose catalytically dead Cas9 (dCas9) for control over gene expression, by tethering dCas9 to a diverse range of transcriptional and epigenetic effectors (see Figures 4–6; also reviewed in Thakore et al., 2016).

### Base Editing Technology

The most common genetic variants associated with human disease in the CNS are point mutations and functional single-nucleotide polymorphisms (SNPs; Nussbaum, 2018). As such, a gene-editing system with the capability to safely, efficiently, and accurately convert single nucleobases has the potential to completely correct many genes implicated in neurodegenerative disease. The creation of a cytosine base-editor (CBE) in David Liu’s lab was the first major advancement towards the development of such tools (Figure 4B). Komor et al. (2016) fused catalytically deficient, or “dead,” Cas9 (dCas9) with rat APOBEC1, a cytosine deaminase enzyme. The resulting complex catalyzes the conversion of all cytosines (Cs) within a 5–6 nucleotide window to uracils (Us); this window ranges from approximately 12–18 nucleotides upstream of the 5’ end of the dCas9’s PAM. The uracil is then read as thymine during replication, completing the C-to-T conversion. However, this intermediate formation of uracil can trigger cellular uracil DNA glycosylase to perform base excision repair, reverting the uracil to cytosine and limiting the base editor’s ability. To combat this problem, a second tool (base editor 2; BE2), was created. It additionally includes the fusion of a uracil glycosylase inhibitor onto dCas9, blocking base excision repair, and significantly increasing the base editor’s efficiency. To further improve BE2, dCas9 was replaced with a Cas9 nickase which cuts only the non-edited strand. Nicking the non-edited strand induces mismatch repair, where the cell preferentially cleaves away the nicked strand and repairs it based on the intact (in this case, edited) strand. This new construct (BE3) was tested in a variety of human cell lines, resulting in the permanent correction of 15–75% of genomic DNA targets. The creation of a single-stranded break did increase the possibility of indel formation from less than 0.1% to approximately 1%; however, this is still a remarkably low rate (Komor et al., 2016).

Since then, the base editing system has been further enhanced. A second copy of the uracil glycosylase inhibitor and a bacteriophage protein called Gam was fused to the nCas9. Gam functions by binding to the free ends of DSBs, thus preventing NHEJ-mediated repair and reducing indel formation. These changes resulted in BE4-Gam, which is characterized by higher base editing efficiency and decreased indel frequency (Komor et al., 2017). However, Gam binding may lead to cell death rather than NHEJ repair, which is unlikely to be appropriate for therapeutic applications. Separately, Koblan et al. (2018) added two Nuclear Localization Signals (NLS) to nCas9 and performed codon-optimization and ancestral sequence reconstruction on APOBEC, yielding BE4max, and ancBE4max. BE4max was then used to efficiently edit two previously challenging to modify disease-relevant SNPs; MPDU1 in human patient-derived fibroblasts and SCN9a intron 6a in mouse neuroblastomas (Koblan et al., 2018). Other researchers have focused on limiting or expanding the cytosine deaminase activity window, and these new constructs allow for C-to-T conversions within a window as short as 3 or as long as 12 nucleotides (Rees and Liu, 2018).

By definition, cytosine base editors catalyze only C-to-T conversions, greatly limiting the range of correctable disease-causing mutations. As such, the creation of an adenosine base editor (ABE), which causes A-to-G conversions, vastly broadens the applicability of base editing (Figure 4C). The first ABE was created by Gaudelli et al. (2017) who fused nickase Cas9 with deoxyadenosine deaminase, which catalyzes the conversion of adenosine to inosine. Similarly to the two-step cytosine editing mechanism, the inosine is then read as guanine during replication, completing the A-to-G conversion. Notably, deoxyadenosine deaminase is not a naturally occurring enzyme and had to be forcefully evolved from the adenosine deaminase TadA, which only recognizes RNA substrates (Gaudelli et al., 2017). This multistep artificial selection process resulted in ABE7, which displayed an average editing efficiency of 53% in HEK293T cells, with an indel formation rate of less than 0.1%. However, a major downside of ABE7 in comparison with its CBE counterparts is incompatibility with Cas9 of any origin other than *Streptococcus pyogenes* (SpCas9). This incompatibility is due to the low DNA-bound residence time of non-SpCas9, coupled with the slow enzymatic rate of deoxyadenosine deaminase. To address this problem, Richter et al. (2020) used phage-assisted-continuous evolution (PACE) and phage-assisted non-continuous evolution (PANCE) methods to enhance the catalytic rate of the deoxyadenosine deaminase enzyme 590-fold, creating ABE8e. ABE8e also displays increased processivity, which is especially beneficial for multiplexed approaches. However, the downside to the new system is an expected increase in Cas9-dependent off-target editing. Similarly, using a modified version of the artificial selection system they established during the creation of ABE7, Gaudelli and colleagues created an array of new 8th generation ABEs, which are characterized by increased activity and editing efficiency, and a broader window of editing. It will be interesting to see whether any 8th gen ABEs or ABE8e can outperform ABE7 *in vivo*, and to what extent. ABE7 has already shown success in an adult mouse model of Duchenne muscular dystrophy, able to correct the *DMB* gene in 17% of myofibers, with no indels or off-targets detected. The 17% rate of cells corrected is highly significant, considering only 4% expression is needed to improve muscle function (Ryu et al., 2018).

### Prime-Editing Technology

Base editing’s profound capabilities are unfortunately limited to C-to-T/G-to-A (CBE) and A-to-G/T-to-C (ABE) base substitutions. This shortcoming inspired David Liu’s lab to develop an ingenious approach to gene editing called prime editing (Anzalone et al., 2019). The protein complex is composed of nCas9 fused with an engineered reverse transcriptase. Importantly, the prime editing guide RNA (pegRNA) differs significantly from regular sgRNAs and plays a major role in the system’s function. The pegRNA acts as both a guide for the nickase Cas9 domain and a template for the fused reverse transcriptase domain (see Figure 5). First, the 5’ end of the pegRNA binds to its DNA target, exposing the noncomplementary strand. The unbound DNA of the “PAM strand” (termed as such because it contains the downstream PAM motif) is then nicked. The very 3’ end of the pegRNA then acts as a PBS, hybridizing with the recently nicked PAM strand. The exposed 3’-OH group on the nicked PAM strand is then extended by reverse transcriptase, using the interior of the pegRNA as its template. The result is two redundant PAM strands, or “flaps”: the edited 3’ flap that was just reverse transcribed from the pegRNA and the unedited 5’ flap. Which of these two flaps hybridizes with the non-PAM strand is theoretically an equilibrium process; in fact, the unedited 5’ flap is thermodynamically favored to hybridize over the edited flap. However, 5’ flaps are also preferentially degraded by cellular endonucleases, which are abundant because of their function in lagging strand synthesis. Thus, the 5’ flap is usually degraded, and the 3’ flap inserted and ligated (see Figure 5). The outcome of this step is a DNA heteroduplex with one edited strand and the other nonedited. The introduction of a nick in the nonedited strand can be accomplished by providing a separate (traditional) sgRNA which guides the prime-editing complex to the unedited strand. The edited strand is thus preferentially used as a template for DNA repair. The addition of the sgRNA represents the latest advancement in the prime-editing system, dubbed PE3. Ideally, the sgRNA should be designed such that it matches the edited strand and not the original, forcing unedited strand nicking to only occur post-edit. This ensures that two nicks are never present at one time, greatly reducing indel formation. This optimal use-case (which is not always possible, due to PAM sequence constraints) also confers a new label: PE3b. PE3b has been shown to support targeted insertions of up to 44 bps, deletions of up to 80 bps, and all 12 types of point mutations, without requiring double-strand breaks or a donor DNA template. Its efficiency in HEK293T cells ranges from approximately 20–50% with 1–10% indel formation. Furthermore, PE3b supports simultaneous combinational edits ranging from 3 bps upstream to 29 bps downstream of the Cas9 PAM motif (Anzalone et al., 2019). In sum, the advantages of prime editing over base editing are numerous: no window of activity removes the possibility of “bystander” mutations, there are less stringent PAM requirements due to the varied length of the RT template, and pegRNA has an approximately 4.4-fold lower off-target editing rate vs. sgRNA. The low off-target rate is due to the need for complementation at Cas9 binding, PBS binding, and RT product complementation for flap resolution. That being said, at the moment base editing offers higher efficiency and lower indel formation, and thus should be used over prime editing whenever possible. Prime editing is still in it is infancy, and it is *in vivo* efficacy is yet to be determined. However, the potential impact on gene editing is enormous, underscored by theoretically being able to correct 89% of known pathogenic mutations and disease-associated genetic variants (Anzalone et al., 2019).

## Epigenetic Regulation by CRISPR/dCas Systems

First, a note on the definition of the term “epigenetics.” In its strictest sense, epigenetics refers to heritable, information-bearing DNA modifications apart from the nucleotide sequence itself (Adli, 2018). The two main types of these epigenetic marks (DNA methylation and histone modifications) will be discussed below. However, we will be using the term “epigenetics” in its more colloquial sense, which more loosely refers to any regulation of gene expression (i.e., transcription) not taking place at the primary sequence level. In the past half-decade, the fusion of catalytically dead Cas9 with various regulatory domains has given researchers unprecedented control over gene expression *in vitro* and *in vivo*, allowing for the therapeutic reprogramming of cell and tissue behavior. Here we will review the current state of dCas9-based epigenetic controllers.

### DNA Methylation

The C^5^ position of DNA-incorporated cytosine can be methylated by DNA methyltransferase enzymes (DNMTs); in mammals, this modification occurs *only* when the cytosine is part of the specific two-base sequence CpG. Cytosine methylation is highly mutagenic; spontaneous deamination of 5-methylcytosine produces thymine, thus converting the CG dinucleotide to TG. Over evolutionary time, the CpG sites which were constitutively methylated have been eliminated from the human genome by precisely this mechanism. The remaining sites, referred to as “CpG islands,” are enriched in the promotor regions of genes, where their methylation causes stable, heritable transcriptional repression (Egger et al., 2004). Furthermore, dysregulation of DNA methylation is the cause of multiple neurodevelopmental disorders, including Fragile X syndrome, in which the expansion of a CGG repeat in the *FMR1* promotor leads to *de novo* DNA methylation and silencing of gene expression (Jin and Warren, 2000), and Rett syndrome, which is caused by mutations in the transcriptional inhibitor methyl-CpG-binding protein 2 (MeCP2), which specifically binds methylated DNA (Amir et al., 1999).

Multiple groups have reported efficient, targeted DNA methylation and gene silencing by fusing dCas9 to the *de novo* DNA methyltransferase enzyme DNMT3A (Liu et al., 2016; McDonald et al., 2016; Vojta et al., 2016). Furthermore, the dCas9-DNMT3A activity can be significantly increased by the additional fusion of the DNMT3A heterodimerization partner DNMT3L (Saunderson et al., 2017; Stepper et al., 2017). The use of DNMT3A has also been combined with the “SunTag” signal amplification system (Huang et al., 2017). In this system, dCas9 is conjugated to a repeating peptide epitope, which then recruits multiple copies of an antibody-effector fusion protein to the desired genomic location. Importantly, Pflueger and colleagues reported that the use of SunTag-DNMT3A resulted in a substantial decrease in off-target DNA methylation compared to a direct dCas-DNMT3A fusion strategy (Pflueger et al., 2018). DNA methyltransferase domains other than DNMT3A have also been fused to dCas9 with similar results, including the prokaryotic DNMT MQ1 (Lei et al., 2017).

Conversely, efficient DNA demethylation has been achieved using dCas9 fusions with the catalytic domain of the methylcytosine dioxygenase TET1 (Choudhury et al., 2016a; Liu et al., 2016). Liu and colleagues evaluated the therapeutic potential of this system by targeting the CGG expansion which causes fragile X syndrome. They found that dCas9-TET1 reduced methylation of the FMR1 promotor and reversed the fragile X-associated loss of the *FMR1* gene product FMRP (Liu et al., 2018). Importantly, the restored expression of FMRP was maintained following the engraftment of *ex vivo* edited cells into mouse brains (Liu et al., 2018). Notably, TET1 has also been employed in conjunction with the SunTag system (Morita et al., 2016).

### Histone Modifications

In nature, DNA does not exist as free strands, but is wrapped around nucleosomes—octamers of histone proteins—like “beads on a string”; the other notable epigenetic marks are applied to these histones rather than DNA itself. Lysine residues in the N-terminal tails of DNA-bound histones are subject to two distinct forms of chemical modification: acetylation and methylation. Histone acetylation, which occurs at multiple lysines resides across histones, neutralizes the lysine’s positive charge, weakening the association of the nucleosome subunits. Generally, this leads to an increase in DNA accessibility and transcriptional activation (Egger et al., 2004). More complex histone methylation generally occurs upstream of acetylation. In contrast to acetylation, methylation of different lysine residues produces profoundly varied, often opposing effects on transcription. Further complicating the process, lysine residues can be mono-, di-, or tri-methylated, which also lead to different downstream effects. The combination of these two factors (and the presence of less common histone modifications) results in what is termed the “histone code.” Briefly, methylation of stimulatory lysines, such as histone 3-lysine 4 (H3K4), causes the recruitment of transcriptional activation complexes, histone acetylation, and an increase in transcription. Conversely, methylation of inhibitory lysines such as H3K9 and H3K27 causes the recruitment of nucleosome-binding proteins, leading to the formation of higher-order chromatin structures and transcriptional silencing. Further complexities of the histone code are beyond the scope of this discussion, but have been excellently reviewed elsewhere (Bannister and Kouzarides, 2011).

CRISPR-based tools have been developed for bidirectional manipulation of both acetylation and methylation. Hilton et al. (2015) showed that a fusion of dCas9 and the catalytic domain of the p300 histone acetyltransferase caused robust, target-specific histone acetylation and gene activation. Conversely, Kwon et al. (2017) showed that a dCas9-histone deacetylase 3 (HDAC3) fusion protein reliably produced target-specific histone deacetylation, although this effect curiously led to opposing transcriptional effects in two different cells lines. To affect methylation, a variety of histone methyltransferase domains have also been fused to dCas. Interestingly, direct methylation of H3K4 by a dCas-PRDM9 fusion was sufficient to cause reactivation of silenced genes (Cano-Rodriguez et al., 2016), but direct methylation of H3K27 (by one of three methyltransferase fusion constructs) was not sufficient for *de novo* gene silencing (O’Geen et al., 2017). Lastly, Kearns et al. (2015) employed a fusion of dCas9 and the histone demethylase LSD1. They found that dCas9-LSD1 is capable of causing targeted loss of H3K4 methylation, which notably caused gene repression only when targeted to enhancer (but not promotor) regions (Kearns et al., 2015).

### Transcriptional Regulators

Remarkably, CRISPR-mediated transcriptional modulation can be achieved while using only catalytically inactive Cas9 and sgRNA. Multiple groups have shown that the mere binding of dCas9 to promoters and other regulatory regions can repress transcription by sterically hindering the RNA polymerase machinery (Gilbert et al., 2013; Larson et al., 2013; Qi et al., 2013); this effect has been dubbed “CRISPR interference” (CRISPRi). Nevertheless, the repressive capacity of the system is vastly improved when dCas9 is linked to a transcriptional repressor domain (TRD). The most commonly used is the Krüppel-associated box (KRAB), a small domain found in ~400 human zinc-finger transcription factors; recruitment of KRAB is associated with methylation of H3K9 and gene silencing (Huntley et al., 2006). Multiple groups have shown that transcriptional inhibition using a dCas9-KRAB fusion protein is vastly superior to CRISPRi using dCas9 alone (Gilbert et al., 2013; Thakore et al., 2015). Furthermore, Yeo et al. (2018) recently demonstrated that dCas9 fused to a bipartite repressor consisting of KRAB and MeCP2 was even more effective than dCas9-KRAB. Interestingly, a homo-dimerizing dCas9 construct delivered with multiple sgRNAs, which causes the direct formation of artificial DNA loops, also had an inhibitory effect on transcription, presumably by promoting assembly of higher-order chromatin structures (Hao et al., 2017). Unsurprisingly, epigenetic activation can also be achieved using CRISPR-derived tools, most often by directly fusing dCas9 to a transcriptional activation domain such as VP64 (Maeder et al., 2013; Perez-Pinera et al., 2013) or a tripartite activation construct such as VPR or VPH (Chavez et al., 2015; Weltner et al., 2018). However, inhibition of toxic risk-factor genes—rather than stimulating expression of a loss-of-function gene—is the primary strategy for the treatment of NDDs (Figure 6). Thus, we will leave further details of CRISPR activation to other capable reviewers (Pickar-Oliver and Gersbach, 2019).

### *In vivo* Applications and Size Constraints

Despite the impressive and rapidly diversifying array of CRISPR/Cas-derived tools, an uncomfortable fact remains. The vast majority of the genome- and epigenome-editing constructs described in the previous sections have only been used *in vitro*. Efficient delivery *in vivo* is a significantly more difficult problem. It must further be noted that all the genome editing tools are relatively large and are currently unable to be packaged into single AAVs. To overcome the significant restraints imposed by AAV’s ~4.7 kb functional packaging capacity, researchers have adopted a clever strategy. A large or multi-component transgene is physically split into two pieces, which are packaged into separate AAV vectors. The resulting AAVs are then co-delivered, and the complete protein is reassembled *in situ* by a split intein—a pair of domains which “splice themselves out,” thus joining two peptide chains end-to-end (Chew et al., 2016; Moreno et al., 2018). Integrase-deficient lentiviral vectors are another appealing option, as they are easily capable of packaging either base-editing tool along with the associated sgRNA and all other required/beneficial transcriptional elements. Furthermore, the large packaging capacity of Lentiviruses may prove critical for the delivery of prime-editors, as the complete PE3 system with all the included elements would not even fit in a dual-AAV system. In their original publication describing prime editing, Anzalone et al. (2019) delivered PE3 along with a reporter construct *via* a dual-Lentivirus system, equivalent to the process described with AAV. Incidentally, our lab has expertise efficiently packaging 10 kb inserts (measured LTR-to-LTR) into Lentiviral vectors. With this in mind, the packaging of the PE3 system in all-in-one lentivirus is theoretically possible, even though with likely lower efficiency. Similarly, an LV vector could easily be configured to package PE2, which confers lower efficiency but also lower indels than PE3, all-in-one. Size restrictions are also critical when working with dCas9-effector complexes, although to a more flexible extent. One common solution when using CRISPR in mice is to simply use a transgenic line stably expressing dCas9 fused to a domain from one of several protein-protein or RNA-protein interaction systems. The complementary domain can then be fused to an epigenetic effector of choice and delivered along with the targeting sgRNA, all of which will fit in a single AAV (Liao et al., 2017; Wangensteen et al., 2018; Zhou et al., 2018). It should be noted that a similar method could be used when using the aforementioned genome editing tools in mice. Lastly, the previously discussed split-intein dual-AAV method has also been used to deliver dCas9-based epigenetic modulators in animal models (Chew et al., 2016; Moreno et al., 2018).

Despite its successes, it must be emphasized that a dual-vector delivery platform has significant caveats. Preps have to be made separately and then combined, meaning twice the viral load must be injected for an equivalent effect compared to an all-in-one system. Furthermore, each target cell must be co-transduced by each vector, or the system fails. However, *in vivo* delivery of epigenetic CRISPR tools in a single AAV is tantalizingly close at hand. In a recent study by Chew et al. (2016), *in vivo* delivery of SaCas9 (which is substantially shorter than the more commonly used SpCas9) fused to the KRAB repressor domain (which contains a mere 45 amino acids) required a second AAV only for delivery of the guide RNA (Thakore et al., 2018); for comparison, the longer SpCas9-KRAB construct (along with sgRNA) easily fits in a single lentiviral vector (Zheng et al., 2018). In a parallel effort to create very small epigenetic modulators, some groups have taken inspiration from the CRISPR system but jettisoned the use of CRISPR itself. Remarkably, Rauch et al. (2019) were able to rationally assemble an active, guide RNA-directed endonuclease out of pre-existing catalytic and RNA-binding domains. This system, dubbed CIRTS, is less than 1/3 the size of SpCas9, and easily able to fit in a single AAV. Although such creative approaches are potentially of great value, CRISPR currently has no competition as the gene-manipulation platform of choice; CIRTS only targets mRNA, and its efficiency pales in comparison to equivalent CRISPR-derived tools. In the coming years, the development of robust CRISPR-based gene editing tools which are capable of being packaged in a single AAV vector will be of the utmost importance. Fortunately, given the amount of scientific talent invested in the advancement of CRISPR/Cas, we do not doubt that single-AAV delivery will soon become commonplace.

## Overview of CRISPR/Cas Systems and Their Use for The Treatment of NDDs

NDDs are defined as any disease that causes the progressive deterioration of nerve cells in the central or peripheral nervous system, a category which naturally encompasses a variety of conditions. However, of the ~7.4 million Americans with an NDD, the vast majority suffer from one of only two: Alzheimer’s disease (AD; 5.4 million) and Parkinson’s disease (PD; 1.5 million; Pal, 2012). As these conditions will be covered in-depth, we must, unfortunately, omit any detailed discussion of other NDDs, the most prominent being multiple sclerosis, which currently affects approximately 400,000 Americans.

AD is a debilitating neurodegenerative disorder characterized by cognitive decline, the risk for which increases significantly with age (reviewed in Gottschalk et al., 2016; Alzheimer’s Dementia, 2020). Phenotypically, AD is characterized by the formation of extracellular plaques of β-amyloid protein (Aβ) and intracellular tangles consisting of the tau protein. To date, the only gene consistently found to be associated with the common, sporadic form of AD (late-onset AD; LOAD) is apolipoprotein E (*APOE*; 2020). APOE was discovered nearly five decades ago (Shore and Shore, 1974), though it took more than two decades to find that APOE has a vital function in the brain (Pitas et al., 1987). Humans have multiple variants of the *APOE* gene (McIntosh et al., 2012), the two most important of which are APOEε3 and APOEε4 (Castellano et al., 2011), while all other animals have only a single APOE isoform (resembling human APOEε3). Only a single amino acid difference exists between APOEε3 (Cys112) and APOEε4 (Arg112). Nevertheless, carrying the APOEε4 variant significantly increases lifetime risk for LOAD, and the presence of two copies is associated with further increased risk (Friedmann, 1976; Alzheimer’s Dementia, 2020) and earlier disease onset (Moskvina et al., 2013; Nussbaum, 2013). We and other groups have suggested that alterations in the expression of APOE in general, and the ε4 isoforms in particular, maybe an important mechanism in the etiology of LOAD (Gottschalk et al., 2016). Therefore, the development of CRISPR/Cas-based therapies targeting APOE and/or APOEε4 expression would offer a valuable epigenetics-based approach for the treatment of LOAD. Below, we will describe current progress and future efforts towards targeting APOE.

Also, we aim for this review to provide a perspective on the etiopathogenesis of PD, which may provide an alternative avenue of research and treatment for the disease. The presence of alpha-synuclein (α-syn) aggregates defines a spectrum of disorders collectively termed synucleinopathies, of which PD is arguably the most well-characterized. Aggregated α-syn is the primary component of Lewy bodies, the defining pathological feature of PD, and point mutations or multiplications in the *SNCA* gene (which expresses α-syn) result in familial PD. The tight link between α-syn expression and PD has led to the hypothesis that α-syn accumulation may produce toxicity through a gain-of-function mechanism. Indeed, misfolding of α-syn leads to the formation of toxic oligomers and beta-pleated sheets, which are thought to impair the proper function of the mitochondria, proteasome, and lysosome-dependent degradation pathways (Poewe et al., 2017). These contribute to neuronal death, mostly within dopaminergic neurons of the substantia nigra pars compacta. This in turn leads to dopamine deficiency in the striatum, which is responsible for the overt symptoms of PD (Poewe et al., 2017).

As elevated levels of α-syn have been implicated in the pathogenesis of PD, targeting SNCA expression levels is an attractive neuroprotective strategy, and manipulations of SNCA expression have demonstrated beneficial effects (reviewed in Tagliafierro and Chiba-Falek, 2016). Several studies have attempted to reduce the expression of α-syn and rescue PD-related phenotypes by directly targeting SNCA mRNA. Flierl et al. (2014) showed that a lentivirus expressing a short hairpin RNA (shRNA) targeting SNCA was capable of rescuing multiple phenotypic abnormalities in SNCA-Tri (triplicated) human neuroprogenitor cells (NPCs), including viability, growth, energy metabolism, and stress resistance (Flierl et al., 2014). Efficient knockdown of SNCA was also reported in a study utilizing small interfering RNA (siRNA), which was injected directly into the monkey substantia nigra (McCormack et al., 2010). A siRNA-based approach also achieved a significant improvement in motor function in a fly model of PD (Takahashi et al., 2015). Notwithstanding these successes, the RNAi approach bears two significant caveats. First, RNAi can affect the expression of genes other than the intended targets, as shown by whole-genome expression profiling after siRNA transfection (Jackson et al., 2003). Second, RNAi does not support the fine resolution of knockdown severity, where tight regulation is needed to achieve a physiological level of SNCA expression (Tagliafierro and Chiba-Falek, 2016). For example, an AAV-siRNA system targeting SNCA caused significant toxicity and a massive loss of nigrostriatal dopaminergic neurons in rat models, inadvertently showing that a *complete* loss of α-syn can cause neurodegeneration (Gorbatyuk et al., 2010).

These examples demonstrate the need for novel therapeutic strategies targeting the regulatory mechanisms controlling SNCA expression, rather than directly targeting the mRNA or the protein, such that precise regulation of α-synuclein levels can be achieved. To this end, our group recently developed a system, comprising an all-in-one lentivirus, for targeted DNA methylation (i.e., epigenome editing) within a regulatory region in SNCA intron 1. This system (dCas9 fused with the catalytic domain of DNMT3A methyltransferase, and associated sgRNA), when delivered to hiPSC-derived dopaminergic neurons from PD patients with SNCA triplications, yielded fine-tuned downregulation of SNCA mRNA and protein levels (Kantor et al., 2018). Furthermore, this effect rescued PD-related cellular phenotypes in these cells, including mitochondrial ROS production and cellular viability (Kantor et al., 2018). These results provide a proof-of-concept validation that DNA hypermethylation at SNCA intron 1 is an effective means of SNCA repression, confirming this general approach as a novel epigenetics-based therapeutic strategy for PD.

While most cases of PD and AD are sporadic, a small subset of both AD and PD cases result from single, causative mutations, which are inherited in a classic Mendelian fashion. These familial forms of AD/PD present earlier in life and are generally very severe. Specifically, early-onset AD is caused mostly by mutations in *APP, PSEN1*, and *PSEN2* (Masters et al., 2015). The pathological beta-amyloid peptide discussed above is a cleavage product of *APP*. Mutations in any of these three genes result in increased AB42/AB40 ratios, and the increase in aggregation-prone AB42 leads to early plaque formation and symptom onset (Masters et al., 2015). In addition to the previously mentioned mutations/multiplications in the *SNCA* gene, autosomal-dominant forms of PD are caused by mutations in leucine repeat kinase 2 (*LRRK2*), and autosomal-recessive PD is caused by mutations in parkin, PTEN-induced putative kinase 1 (*PINK1*), and Daisuke-Junko-1 (*DJ-1*, Scott et al., 2017). These and other genes involved in the etiology of PD, including FBX07, ATP13A2, DNAJC1, PLA2G634, SYNJ1, VPS35, eiF4G1, and CHCHD2, are reviewed elsewhere (Scott et al., 2017).

While the devil is always in the details, for these patients the overall therapeutic strategy is obvious: simply correct the causative genetic mutation, using base editing if it is a valid target, or prime editing if not. This strategy is very similar to that which would be appropriate for any other CNS disease caused by a single, correctable genetic mutation. The first proof-of-concept study validating a base-editing approach *in vivo* on post-mitotic sensory cells came from David Liu’s lab (Yeh et al., 2018). The authors used base editing to install an S33F mutation in the β-catenin gene, successfully upregulating Wnt signaling (which is involved in mitosis of cochlear supporting cells and cellular reprogramming). In contrast, delivery of nuclease-active Cas9 to install the S33F mutation *via* HDR did not produce a measurable induction of Wnt signaling (Yeh et al., 2018). Two years earlier, the same lab validated the base-editing system *in vitro* by converting APOEε4 into APOEε3 in immortalized mouse astrocytes, in which the endogenous APOE gene was replaced by human APOEε4. In this study, Komor et al. (2016) transfected the CBE system and an appropriate sgRNA placing the target cytosine at position 5 relative to a downstream PAM, resulting in a conversion rate of up to 10%. Indeed, the generation of APOEε3/4 iPSC lines *via* base-pair editing has become a routine task for many labs and is now offered as a service from biotech companies. As an example of this technique, BE4max was used to generate base-edited isogenic hiPSC lines using a transient reporter for editing enrichment (BIG-TREE). Relevantly, the researchers efficiently generated multiple clonal lines bearing different APOE genotypes, with an astonishing 90% of isolated clones being edited (Brookhouser et al., 2020).

Base-editing technology has become available only very recently. Interestingly, an older editing technology—the zinc-finger nuclease (ZFN) system—was recently applied to generate isogenic APOEε3 and ε4 iPSC lines, by Wang and coworkers (Wang et al., 2018). Using human neurons derived from the isogenic iPSCs, they showed that APOEε3-expressing neurons had higher levels of tau phosphorylation, unrelated to their increased production of Aβ peptides. Further, they displayed GABAergic neuron degeneration. Gene editing to APOEε3 rescued these phenotypes, indicating a specific effect of APOEε3. Crucially, the authors also reported that APOE knockout neurons behave similarly to those expressing APOEε3, and that re-introduction of APOEε3 restored the pathological phenotypes associated with AD; these results suggest that APOEε4 has a toxic gain-of-function effect.

As mentioned above, to best of our knowledge, base/prime editing systems have not yet been applied to animal models of familial AD or PD, but recent days have seen groundbreaking results targeting other CNS diseases. For example, Levy et al. (2020) recently applied a dual-AAV9 system to deliver SpCas9-CBE to the brain of a mouse model of Niemann-Pick disease type C. They successfully edited approximately 48% of cortical cells (mixed cell types from unsorted tissue), and 0.3% of cerebellar cells, with minimal indel formation, off-targets, or bystander mutations. The result was an increase in surviving Purkinje neurons and an increase in lifespan of about 10%. Even more recently, Li et al. (2020) used an ABE derived from the very short *Campylobacter jejuni* Cas9 (CjCas9) to correct an oncogene-activating mutation in the *TERT* gene promotor, which occurs in glioblastoma and many other cancer types. Impressively, localized intracranial injection of a pair of AAVs expressing CjCas9-ABE (and its associated sgRNA, respectively) was capable of arresting the growth of *TERT* mutation-driven gliomas (Li et al., 2020). Notably, compared to many “editable” diseases (particularly developmental disorders) treating familial NDDs with base/prime editing would have at least one major advantage. Unlike inherited developmental disorders, the symptoms of familial NDDs present (relatively) late in life. Thus, viral gene therapy could plausibly be administered until adolescence/adulthood, as opposed to requiring delivery during infancy or earlier.

All that being said, the vast majority of both AD and PD cases are not of the early-onset type. Unlike familial cases, the etiologies of late-onset AD/PD are quite complex, being driven by an intricate web of generally low-impact genetic and environmental risk factors. Unfortunately, attempts to identify unifying pathogenic mechanisms based on the genetics of the familial forms have had mixed results, at best. One of the strongest pieces of evidence for the “amyloid hypothesis” of Alzheimer’s pathogenesis is the existence of familial AD caused by mutations in APP, PSEN1, and PSEN2. Regardless, several AD therapies based on this hypothesis have recently suffered devastating failures in clinical trials for late-onset AD (Mullard, 2019). Importantly, many of these therapeutics very effectively cleared beta-amyloid plaques from the brain, yet patients saw no improvements, nor delays in disease progression. If the mechanisms underlying these diseases can be fully elucidated, the potential for prevention or reversal of progression and pathophysiology will increase considerably (Gottschalk et al., 2016; Tagliafierro and Chiba-Falek, 2016; Kampmann, 2017; Lutz et al., 2020).

As far as therapeutic options are concerned, safe, permanent “knockdowns” can be achieved using base editing. For example, a mouse model of ALS was treated by using BE3 to introduce a nonsense mutation in SOD1, resulting in prolonged survival and slowed disease progression, even in adult mice (Lim et al., 2020). However, the use of dCas9-based epigenetic effectors similar to those developed in our laboratory (Tagliafierro and Chiba-Falek, 2016) provides an additional, complementary approach to gene repression.

A major concern over viral vectors is lingering uncertainty over their safety profile. Although progress has been made to reduce the toxicity of viruses by directed evolution and engineering, most viruses that infect cells and deliver genes will inevitably integrate their genetic elements into the host genome. These elements can pose long-term safety risks. Furthermore, viral vectors possess a significant risk associated with their ability to activate deleterious immune responses. Last but not least, a drawback of viruses is that their production is labor-intensive, and clinical applications are expensive because each step of clinical-grade viral vector manufacturing must strictly comply with good manufacturing practices (GMP).

While we focus here on factors most relevant to viral vector design and production, we would be remiss if we did not note other novel delivery technologies being developed in parallel. Recently, Park et al. (2019) used a “traditional CRISPR” strategy (i.e., creation of disruptive indels by nuclease-active Cas9) to successfully alleviate behavioral deficits in two mouse models of familial AD. These constructs were delivered not by a viral vector, but *via* nano complexes composed of a synthetic, amphiphilic peptide (Arg_7_-Leu_10_). This is a highly innovative approach—reminiscent of traditional chemical transfection—which hopefully has therapeutic potential as well.

A few final points are worthy of discussion before concluding. First, future gene therapies for early- and late-onset AD/PD may be quite different. Second, and critically, the small size of many of the epigenetic repressor modules would make packaging into a single all-in-one AAV delivery vector relatively straightforward. Lastly, both AAV-based delivery and the epigenetic strategy itself carry significant advantages. Epigenetic approaches have the benefit of never physically modifying the DNA target, ruling out an entire class of potential off-target effects. And as mentioned above, AAV vectors have an unparalleled safety profile. Indeed, AAVs are the only delivery vehicle approved to administer CRISPR-based therapeutics to humans (Wang et al., 2020). As such, while developing AAV-compatible epigenetic therapies for late-onset AD and PD is sure to be a challenge, it is a worthy one, with the promise of lasting clinical reward.

## Author Contributions

JR and MM wrote the review, with guidance and edits from OC-F and BK.

## Conflict of Interest

The authors declare that the research was conducted in the absence of any commercial or financial relationships that could be construed as a potential conflict of interest.

## Footnotes

^1^http://abedia.com/wiley/index.html

## References

[B1] Adli M. (2018). The CRISPR tool kit for genome editing and beyond. Nat. Commun. 9:1911. 10.1038/s41467-018-04252-2 29765029 PMC5953931

[B2] Alzheimer’s Dementia. (2020). 2020 Alzheimer’s disease facts and figures. Alzheimers Dement. 16, 391–460. 10.1201/b15134-432157811

[B3] Amir R. E. Van den Veyver I. B. Wan M. Tran C. Q. Francke U. Zoghbi H. Y. (1999). Rett syndrome is caused by mutations in X-linked MECP2, encoding methyl-CpG-binding protein 2. Nat. Genet. 23, 185–188. 10.1038/13810 10508514

[B4] Anders C. Niewoehner O. Duerst A. Jinek M. (2014). Structural basis of PAM-dependent target DNA recognition by the Cas9 endonuclease. Nature 513, 569–573. 10.1038/nature13579 25079318 PMC4176945

[B5] Anzalone A. V. Randolph P. B. Davis J. R. Sousa A. A. Koblan L. W. Levy J. M.. (2019). Search-and-replace genome editing without double-strand breaks or donor DNA. Nature 576, 149–157. 10.1038/s41586-019-1711-4 31634902 PMC6907074

[B7] Azzouz M. Martin-Rendon E. Barber R. D. Mitrophanous K. A. Carter E. E. Rohll J. B.. (2002). Multicistronic lentiviral vector-mediated striatal gene transfer of aromatic L-amino acid decarboxylase, tyrosine hydroxylase and GTP cyclohydrolase I induces sustained transgene expression, dopamine production and functional improvement in a rat model of Parkinson’s disease. J. Neurosci. 22, 10302–10312. 10.1523/JNEUROSCI.22-23-10302.2002 12451130 PMC6758736

[B8] Bannister A. J. Kouzarides T. (2011). Regulation of chromatin by histone modifications. Cell Res. 21, 381–395. 10.1038/cr.2011.22 21321607 PMC3193420

[B9] Barrangou R. Fremaux C. Deveau H. Richards M. Boyaval P. Moineau S.. (2007). CRISPR provides acquired resistance against viruses in prokaryotes. Science 315, 1709–1712. 10.1126/science.1138140 17379808

[B10] Bartlett J. S. Wilcher R. Samulski R. J. (2000). Infectious entry pathway of adeno-associated virus and adeno-associated virus vectors. J. Virol. 74, 2777–2785. 10.1128/jvi.74.6.2777-2785.2000 10684294 PMC111768

[B11] Bayer M. Kantor B. Cockrell A. Ma H. Zeithaml B. Li X.. (2008). A large U3 deletion causes increased *in vivo* expression from a nonintegrating lentiviral vector. Mol. Ther. 16, 1968–1976. 10.1038/mt.2008.199 18797449 PMC2587457

[B12] Blaese R. M. Culver K. W. Miller A. D. Carter C. S. Fleisher T. Clerici M.. (1995). T lymphocyte-directed gene therapy for ADA-SCID: initial trial results after 4 years. Science 270, 475–480. 10.1126/science.270.5235.475 7570001

[B13] Blömer U. Naldini L. Kafri T. Trono D. Verma I. M. Gage F. H. (1997). Highly efficient and sustained gene transfer in adult neurons with a lentivirus vector. J. Virol. 71, 6641–6649. 10.1128/jvi.71.9.6641-6649.1997 9261386 PMC191942

[B14] Brookhouser N. Tekel S. J. Standage-Beier K. Nguyen T. Schwarz G. Wang X.. (2020). BIG-TREE: base-edited isogenic hPSC line generation using a transient reporter for editing enrichment. Stem Cell Reports 14, 184–191. 10.1016/j.stemcr.2019.12.013 32004495 PMC7013208

[B15] Buchow H. D. Tschachler E. Gallo R. C. Reitz M. Jr. (1989). HIV-I replication requires an intact integrase reading frame. Haematol. Blood Transfus. 32, 402–405. 10.1007/978-3-642-74621-5_68 2560448

[B16] Burger C. Gorbatyuk O. S. Velardo M. J. Peden C. S. Williams P. Zolotukhin S.. (2004). Recombinant AAV viral vectors pseudotyped with viral capsids from serotypes 1, 2 and 5 display differential efficiency and cell tropism after delivery to different regions of the central nervous system. Mol. Ther. 10, 302–317. 10.1016/j.ymthe.2004.05.024 15294177

[B17] Cano-Rodriguez D. Gjaltema R. A. Jilderda L. J. Jellema P. Dokter-Fokkens J. Ruiters M. H.. (2016). Writing of H3K4Me3 overcomes epigenetic silencing in a sustained but context-dependent manner. Nat. Commun. 7:12284. 10.1038/ncomms12284 27506838 PMC4987519

[B18] Casini A. Olivieri M. Petris G. Montagna C. Reginato G. Maule G.. (2018). A highly specific SpCas9 variant is identified by *in vivo* screening in yeast. Nat. Biotechnol. 36, 265–271. 10.1038/nbt.4066 29431739 PMC6066108

[B19] Castellano J. M. Kim J. Stewart F. R. Jiang H. DeMattos R. B. Patterson B. W.. (2011). Human ApoE isoforms differentially regulate brain amyloid-β peptide clearance. Sci. Transl. Med. 3:89ra57. 10.1126/scitranslmed.3002156 21715678 PMC3192364

[B20] Castle M. J. Turunen H. T. Vandenberghe L. H. Wolfe J. H. (2016). Controlling AAV tropism in the nervous system with natural and engineered capsids. Methods Mol. Biol. 1382, 133–149. 10.1007/978-1-4939-3271-9_10 26611584 PMC4993104

[B21] Cearley C. N. Wolfe J. H. (2006). Transduction characteristics of adeno-associated virus vectors expressing cap serotypes 7, 8, 9, and Rh10 in the mouse brain. Mol. Ther. 13, 528–537. 10.1016/j.ymthe.2005.11.015 16413228

[B22] Chan K. Y. Jang M. J. Yoo B. B. Greenbaum A. Ravi N. Wu W. L.. (2017). Engineered AAVs for efficient noninvasive gene delivery to the central and peripheral nervous systems. Nat. Neurosci. 20, 1172–1179. 10.1038/nn.4593 28671695 PMC5529245

[B23] Chavez A. Scheiman J. Vora S. Pruitt B. W. Tuttle M. Iyer E. P. R.. (2015). Highly efficient Cas9-mediated transcriptional programming. Nat. Methods 12, 326–328. 10.1038/nmeth.3312 25730490 PMC4393883

[B24] Chen J. S. Dagdas Y. S. Kleinstiver B. P. Welch M. M. Sousa A. A. Harrington L. B.. (2017). Enhanced proofreading governs CRISPR-Cas9 targeting accuracy. Nature 550, 407–410. 10.1038/nature24268 28931002 PMC5918688

[B25] Chew W. L. Tabebordbar M. Cheng J. K. Mali P. Wu E. Y. Ng A. H.. (2016). A multifunctional AAV-CRISPR-Cas9 and its host response. Nat. Methods 13, 868–874. 10.1038/nmeth.3993 27595405 PMC5374744

[B26] Choudhury S. R. Cui Y. Lubecka K. Stefanska B. Irudayaraj J. (2016a). CRISPR-dCas9 mediated TET1 targeting for selective DNA demethylation at BRCA1 promoter. Oncotarget 7, 46545–46556. 10.18632/oncotarget.10234 27356740 PMC5216816

[B27] Choudhury S. R. Harris A. F. Cabral D. J. Keeler A. M. Sapp E. Ferreira J. S.. (2016b). Widespread central nervous system gene transfer and silencing after systemic delivery of novel AAV-AS vector. Mol. Ther. 24, 726–735. 10.1038/mt.2015.231 26708003 PMC4886933

[B28] Cockrell A. S. Ma H. Fu K. McCown T. J. Kafri T. (2006). A trans-lentiviral packaging cell line for high-titer conditional self-inactivating HIV-1 vectors. Mol. Ther. 14, 276–284. 10.1016/j.ymthe.2005.12.015 16516556

[B29] Coffin J. M. Hughes S. H. Varmus H. E. (Eds). (1997). “The interactions of retroviruses and their hosts,” in Retroviruses, (New York, NY: Cold Spring Harbor), 335–341. 21433350

[B30] Colicelli J. Goff S. P. (1985). Mutants and pseudorevertants of Moloney murine leukemia virus with alterations at the integration site. Cell 42, 573–580. 10.1016/0092-8674(85)90114-x 4028161

[B31] Consiglio A. Quattrini A. Martino S. Bensadoun J. C. Dolcetta D. Trojani A.. (2001). *In vivo* gene therapy of metachromatic leukodystrophy by lentiviral vectors: correction of neuropathology and protection against learning impairments in affected mice. Nat. Med. 7, 310–316. 10.1038/85454 11231629

[B32] Craigie R. Fujiwara T. Bushman F. (1990). The IN protein of Moloney murine leukemia virus processes the viral DNA ends and accomplishes their integration *in vitro*. Cell 62, 829–837. 10.1016/0092-8674(90)90126-y 2167180

[B33] Cronin J. Zhang X. Y. Reiser J. (2005). Altering the tropism of lentiviral vectors through pseudotyping. Curr. Gene Ther. 5, 387–398. 10.2174/1566523054546224 16101513 PMC1368960

[B34] Deltcheva E. Chylinski K. Sharma C. M. Gonzales K. Chao Y. Pirzada Z. A.. (2011). CRISPR RNA maturation by trans-encoded small RNA and host factor RNase III. Nature 471, 602–607. 10.1038/nature09886 21455174 PMC3070239

[B35] Deverman B. E. Pravdo P. L. Simpson B. P. Kumar S. R. Chan K. Y. Banerjee A.. (2016). Cre-dependent selection yields AAV variants for widespread gene transfer to the adult brain. Nat. Biotechnol. 34, 204–209. 10.1038/nbt.3440 26829320 PMC5088052

[B36] Deyle D. R. Russell D. W. (2009). Adeno-associated virus vector integration. Curr. Opin. Mol. Ther. 11, 442–447. 19649989 PMC2929125

[B37] Duan D. Sharma P. Yang J. Yue Y. Dudus L. Zhang Y.. (1998). Circular intermediates of recombinant adeno-associated virus have defined structural characteristics responsible for long-term episomal persistence in muscle tissue. J. Virol. 72, 8568–8577. 10.1128/jvi.72.11.8568-8577.1998 9765395 PMC110267

[B38] Dull T. Zufferey R. Kelly M. Mandel R. J. Nguyen M. Trono D.. (1998). A third-generation lentivirus vector with a conditional packaging system. J. Virol. 72, 8463–8471. 10.1128/jvi.72.11.8463-8471.1998 9765382 PMC110254

[B39] Duque S. Joussemet B. Riviere C. Marais T. Dubreil L. Douar A. M.. (2009). Intravenous administration of self-complementary AAV9 enables transgene delivery to adult motor neurons. Mol. Ther. 17, 1187–1196. 10.1038/mt.2009.71 19367261 PMC2835208

[B40] Egger G. Liang G. Aparicio A. Jones P. A. (2004). Epigenetics in human disease and prospects for epigenetic therapy. Nature 429, 457–463. 10.1038/nature02625 15164071

[B41] Engelman A. Englund G. Orenstein J. M. Martin M. A. Craigie R. (1995). Multiple effects of mutations in human immunodeficiency virus type 1 integrase on viral replication. J. Virol. 69, 2729–2736. 10.1128/jvi.69.5.2729-2736.1995 7535863 PMC188965

[B42] Federici T. Taub J. S. Baum G. R. Gray S. J. Grieger J. C. Matthews K. A.. (2012). Robust spinal motor neuron transduction following intrathecal delivery of AAV9 in pigs. Gene Ther. 19, 852–859. 10.1038/gt.2011.130 21918551

[B43] Ferrari F. K. Samulski T. Shenk T. Samulski R. J. (1996). Second-strand synthesis is a rate-limiting step for efficient transduction by recombinant adeno-associated virus vectors. J. Virol. 70, 3227–3234. 10.1128/jvi.70.5.3227-3234.1996 8627803 PMC190186

[B44] Flierl A. Oliveira L. M. Falomir-Lockhart L. J. Mak S. K. Hesley J. Soldner F.. (2014). Higher vulnerability and stress sensitivity of neuronal precursor cells carrying an α-synuclein gene triplication. PLoS One 9:e112413. 10.1371/journal.pone.0112413 25390032 PMC4229205

[B45] Foust K. D. Flotte T. R. Reier P. J. Mandel R. J. (2008). Recombinant adeno-associated virus-mediated global anterograde delivery of glial cell line-derived neurotrophic factor to the spinal cord: comparison of rubrospinal and corticospinal tracts in the rat. Hum. Gene Ther. 19, 71–82. 10.1089/hum.2007.104 18072858

[B46] Friedmann T. (1976). The future for gene therapy—a reevaluation. Ann. N Y Acad. Sci. 265, 141–152. 10.1111/j.1749-6632.1976.tb29328.x 1066080

[B47] Gao G. Vandenberghe L. H. Wilson J. M. (2005). New recombinant serotypes of AAV vectors. Curr. Gene Ther. 5, 285–297. 10.2174/1566523054065057 15975006

[B48] Gaudelli N. M. Komor A. C. Rees H. A. Packer M. S. Badran A. H. Bryson D. I.. (2017). Programmable base editing of A*T to G*C in genomic DNA without DNA cleavage. Nature 551, 464–471. 10.1038/nature24644 29160308 PMC5726555

[B49] Gilbert L. A. Larson M. H. Morsut L. Liu Z. Brar G. A. Torres S. E.. (2013). CRISPR-mediated modular RNA-guided regulation of transcription in eukaryotes. Cell 154, 442–451. 10.1016/j.cell.2013.06.044 23849981 PMC3770145

[B50] Ginn S. L. Amaya A. K. Alexander I. E. Edelstein M. Abedi M. R. (2018). Gene therapy clinical trials worldwide to 2017: an update. J. Gene Med. 20:e3015. 10.1002/jgm.3015 29575374

[B51] Gorbatyuk O. S. Li S. Nash K. Gorbatyuk M. Lewin A. S. Sullivan L. F.. (2010). *In vivo* RNAi-mediated α-synuclein silencing induces nigrostriatal degeneration. Mol. Ther. 18, 1450–1457. 10.1038/mt.2010.115 20551914 PMC2927065

[B52] Gottschalk W. K. Mihovilovic M. Roses A. D. Chiba-Falek O. (2016). The role of upregulated APOE in Alzheimer’s disease etiology. J. Alzheimers Dis. Parkinsonism 6:209. 10.4172/2161-0460.1000209 27104063 PMC4836841

[B53] Gray S. J. Nagabhushan Kalburgi S. McCown T. J. Jude Samulski R. (2013). Global CNS gene delivery and evasion of anti-AAV-neutralizing antibodies by intrathecal AAV administration in non-human primates. Gene Ther. 20, 450–459. 10.1038/gt.2012.101 23303281 PMC3618620

[B54] Gray S. J. Woodard K. T. Samulski R. J. (2010). Viral vectors and delivery strategies for CNS gene therapy. Ther. Deliv. 1, 517–534. 10.4155/tde.10.50 22833965 PMC4509525

[B55] Grimm D. Lee J. S. Wang L. Desai T. Akache B. Storm T. A.. (2008). *In vitro* and *in vivo* gene therapy vector evolution *via* multispecies interbreeding and retargeting of adeno-associated viruses. J. Virol. 82, 5887–5911. 10.1128/JVI.00254-08 18400866 PMC2395137

[B56] Haapaniemi E. Botla S. Persson J. Schmierer B. Taipale J. (2018). CRISPR-Cas9 genome editing induces a p53-mediated DNA damage response. Nat. Med. 24, 927–930. 10.1038/s41591-018-0049-z 29892067

[B57] Hao N. Shearwin K. E. Dodd I. B. (2017). Programmable DNA looping using engineered bivalent dCas9 complexes. Nat. Commun. 8:1628. 10.1038/s41467-017-01873-x 29158476 PMC5696343

[B58] Haurigot V. Bosch F. (2013). Toward a gene therapy for neurological and somatic MPSIIIA. Rare Dis. 1:e27209. 10.4161/rdis.27209 25003015 PMC3927492

[B59] Hester M. E. Foust K. D. Kaspar R. W. Kaspar B. K. (2009). AAV as a gene transfer vector for the treatment of neurological disorders: novel treatment thoughts for ALS. Curr. Gene Ther. 9, 428–433. 10.2174/156652309789753383 19860657

[B60] Hilton I. B. D’Ippolito A. M. Vockley C. M. Thakore P. I. Crawford G. E. Reddy T. E.. (2015). Epigenome editing by a CRISPR-Cas9-based acetyltransferase activates genes from promoters and enhancers. Nat. Biotechnol. 33, 510–517. 10.1038/nbt.3199 25849900 PMC4430400

[B61] Hirano H. Gootenberg J. S. Horii T. Abudayyeh O. O. Kimura M. Hsu P. D.. (2016). Structure and engineering of francisella novicida Cas9. Cell 164, 950–961. 10.1016/j.cell.2016.01.039 26875867 PMC4899972

[B62] Hordeaux J. Wang Q. Katz N. Buza E. L. Bell P. Wilson J. M. (2018). The neurotropic properties of AAV-PHP.B are limited to C57BL/6J mice. Mol. Ther. 26, 664–668. 10.1016/j.ymthe.2018.01.018 29428298 PMC5911151

[B63] Hsu P. D. Lander E. S. Zhang F. (2014). Development and applications of CRISPR-Cas9 for genome engineering. Cell 157, 1262–1278. 10.1016/j.cell.2014.05.010 24906146 PMC4343198

[B64] Hu J. H. Miller S. M. Geurts M. H. Tang W. Chen L. Sun N.. (2018). Evolved Cas9 variants with broad PAM compatibility and high DNA specificity. Nature 556, 57–63. 10.1038/nature26155 29512652 PMC5951633

[B65] Huang Y. H. Su J. Lei Y. Brunetti L. Gundry M. C. Zhang X.. (2017). DNA epigenome editing using CRISPR-Cas SunTag-directed DNMT3A. Genome Biol. 18:176. 10.1186/s13059-017-1306-z 28923089 PMC5604343

[B66] Huntley S. Baggott D. M. Hamilton A. T. Tran-Gyamfi M. Yang S. Kim J.. (2006). A comprehensive catalog of human KRAB-associated zinc finger genes: insights into the evolutionary history of a large family of transcriptional repressors. Genome Res. 16, 669–677. 10.1101/gr.4842106 16606702 PMC1457042

[B67] Ihry R. J. Worringer K. A. Salick M. R. Frias E. Ho D. Theriault K.. (2018). p53 inhibits CRISPR-Cas9 engineering in human pluripotent stem cells. Nat. Med. 24, 939–946. 10.1038/s41591-018-0050-6 29892062

[B68] Jackson A. L. Bartz S. R. Schelter J. Kobayashi S. V. Burchard J. Mao M.. (2003). Expression profiling reveals off-target gene regulation by RNAi. Nat. Biotechnol. 21, 635–637. 10.1038/nbt831 12754523

[B69] Jakobsson J. Ericson C. Jansson M. Björk E. Lundberg C. (2003). Targeted transgene expression in rat brain using lentiviral vectors. J. Neurosci. Res. 73, 876–885. 10.1002/jnr.10719 12949915

[B70] Jin P. Warren S. T. (2000). Understanding the molecular basis of fragile X syndrome. Hum. Mol. Genet. 9, 901–908. 10.1093/hmg/9.6.901 10767313

[B71] Jinek M. Chylinski K. Fonfara I. Hauer M. Doudna J. A. Charpentier E. (2012). A programmable dual-RNA-guided DNA endonuclease in adaptive bacterial immunity. Science 337, 816–821. 10.1126/science.1225829 22745249 PMC6286148

[B72] Kafri T. Blömer U. Peterson D. A. Gage F. H. Verma I. M. (1997). Sustained expression of genes delivered directly into liver and muscle by lentiviral vectors. Nat. Genet. 17, 314–317. 10.1038/ng1197-314 9354796

[B73] Kampmann M. (2017). A CRISPR approach to neurodegenerative diseases. Trends Mol. Med. 23, 483–485. 10.1016/j.molmed.2017.04.003 28478951 PMC5604856

[B74] Kantor B. Bailey R. M. Wimberly K. Kalburgi S. N. Gray S. J. (2014a). Methods for gene transfer to the central nervous system. Adv. Genet. 87, 125–197. 10.1016/B978-0-12-800149-3.00003-2 25311922 PMC4519829

[B77] Kantor B. McCown T. Leone P. Gray S. J. (2014b). Clinical applications involving CNS gene transfer. Adv. Genet. 87, 71–124. 10.1016/b978-0-12-800149-3.00002-0 25311921 PMC4518844

[B75] Kantor B. Bayer M. Ma H. Samulski J. Li C. McCown T.. (2011). Notable reduction in illegitimate integration mediated by a PPT-deleted, nonintegrating lentiviral vector. Mol. Ther. 19, 547–556. 10.1038/mt.2010.277 21157436 PMC3048187

[B76] Kantor B. Ma H. Webster-Cyriaque J. Monahan P. E. Kafri T. (2009). Epigenetic activation of unintegrated HIV-1 genomes by gut-associated short chain fatty acids and its implications for HIV infection. Proc. Natl. Acad. Sci. U S A 106, 18786–18791. 10.1073/pnas.0905859106 19843699 PMC2773968

[B78] Kantor B. Tagliafierro L. Gu J. Zamora M. E. Ilich E. Grenier C.. (2018). Downregulation of SNCA expression by targeted editing of DNA methylation: a potential strategy for precision therapy in PD. Mol. Ther. 26, 2638–2649. 10.1016/j.ymthe.2018.08.019 30266652 PMC6224806

[B79] Kearns N. A. Pham H. Tabak B. Genga R. M. Silverstein N. J. Garber M.. (2015). Functional annotation of native enhancers with a Cas9-histone demethylase fusion. Nat. Methods 12, 401–403. 10.1038/nmeth.3325 25775043 PMC4414811

[B80] Keiser N. W. Yan Z. Zhang Y. Lei-Butters D. C. Engelhardt J. F. (2011). Unique characteristics of AAV1, 2, and 5 viral entry, intracellular trafficking and nuclear import define transduction efficiency in HeLa cells. Hum. Gene Ther. 22, 1433–1444. 10.1089/hum.2011.044 21574868 PMC3225038

[B81] Klein R. L. Muir D. King M. A. Peel A. L. Zolotukhin S. Möller J. C.. (1999). Long-term actions of vector-derived nerve growth factor or brain-derived neurotrophic factor on choline acetyltransferase and Trk receptor levels in the adult rat basal forebrain. Neuroscience 90, 815–821. 10.1016/s0306-4522(98)00537-5 10218782

[B82] Kleinstiver B. P. Pattanayak V. Prew M. S. Tsai S. Q. Nguyen N. T. Zheng Z.. (2016). High-fidelity CRISPR-Cas9 nucleases with no detectable genome-wide off-target effects. Nature 529, 490–495. 10.1038/nature16526 26735016 PMC4851738

[B83] Kleinstiver B. P. Prew M. S. Tsai S. Q. Topkar V. V. Nguyen N. T. Zheng Z.. (2015). Engineered CRISPR-Cas9 nucleases with altered PAM specificities. Nature 523, 481–485. 10.1038/nature14592 26098369 PMC4540238

[B84] Koblan L. W. Doman J. L. Wilson C. Levy J. M. Tay T. Newby G. A.. (2018). Improving cytidine and adenine base editors by expression optimization and ancestral reconstruction. Nat. Biotechnol. 36, 843–846. 10.1038/nbt.4172 29813047 PMC6126947

[B85] Kocak D. D. Josephs E. A. Bhandarkar V. Adkar S. S. Kwon J. B. Gersbach C. A. (2019). Increasing the specificity of CRISPR systems with engineered RNA secondary structures. Nat. Biotechnol. 37, 657–666. 10.1038/s41587-019-0095-1 30988504 PMC6626619

[B86] Komor A. C. Badran A. H. Liu D. R. (2018). Editing the genome without double-stranded DNA breaks. ACS Chem. Biol. 13, 383–388. 10.1021/acschembio.7b00710 28957631 PMC5891729

[B87] Komor A. C. Kim Y. B. Packer M. S. Zuris J. A. Liu D. R. (2016). Programmable editing of a target base in genomic DNA without double-stranded DNA cleavage. Nature 533, 420–424. 10.1038/nature17946 27096365 PMC4873371

[B88] Komor A. C. Zhao K. T. Packer M. S. Gaudelli N. M. Waterbury A. L. Koblan L. W.. (2017). Improved base excision repair inhibition and bacteriophage Mu Gam protein yields C:G-to-T:A base editors with higher efficiency and product purity. Sci. Adv. 3:eaao4774. 10.1126/sciadv.aao4774 28875174 PMC5576876

[B89] Koonin E. V. Makarova K. S. Zhang F. (2017). Diversity, classification and evolution of CRISPR-Cas systems. Curr. Opin. Microbiol. 37, 67–78. 10.1016/j.mib.2017.05.008 28605718 PMC5776717

[B90] Körbelin J. Dogbevia G. Michelfelder S. Ridder D. A. Hunger A. Wenzel J.. (2016). A brain microvasculature endothelial cell-specific viral vector with the potential to treat neurovascular and neurological diseases. EMBO Mol. Med. 8, 609–625. 10.15252/emmm.201506078 27137490 PMC4888852

[B91] Kotin R. M. Siniscalco M. Samulski R. J. Zhu X. D. Hunter L. Laughlin C. A.. (1990). Site-specific integration by adeno-associated virus. Proc. Natl. Acad. Sci. U S A 87, 2211–2215. 10.1073/pnas.87.6.2211 2156265 PMC53656

[B92] Kronenberg S. Kleinschmidt J. A. Böttcher B. (2001). Electron cryo-microscopy and image reconstruction of adeno-associated virus type 2 empty capsids. EMBO Rep. 2, 997–1002. 10.1093/embo-reports/kve234 11713191 PMC1084133

[B93] Kulcsár P. I. Talas A. Huszar K. Ligeti Z. Toth E. Weinhardt N.. (2017). Crossing enhanced and high fidelity SpCas9 nucleases to optimize specificity and cleavage. Genome Biol. 18:190. 10.1186/s13059-017-1318-8 28985763 PMC6389135

[B94] Kwon D. Y. Zhao Y. T. Lamonica J. M. Zhou Z. (2017). Locus-specific histone deacetylation using a synthetic CRISPR-Cas9-based HDAC. Nat. Commun. 8:15315. 10.1038/ncomms15315 28497787 PMC5437308

[B95] Larson M. H. Gilbert L. A. Wang X. Lim W. A. Weissman J. S. Qi L. S. (2013). CRISPR interference (CRISPRi) for sequence-specific control of gene expression. Nat. Protoc. 8, 2180–2196. 10.1038/nprot.2013.132 24136345 PMC3922765

[B96] Leavitt A. D. Rose R. B. Varmus H. E. (1992). Both substrate and target oligonucleotide sequences affect *in vitro* integration mediated by human immunodeficiency virus type 1 integrase protein produced in Saccharomyces cerevisiae. J. Virol. 66, 2359–2368. 10.1128/jvi.66.4.2359-2368.1992 1548767 PMC289031

[B97] Lei Y. Zhang X. Su J. Jeong M. Gundry M. C. Huang Y. H.. (2017). Targeted DNA methylation *in vivo* using an engineered dCas9-MQ1 fusion protein. Nat. Commun. 8:16026. 10.1038/ncomms16026 28695892 PMC5508226

[B98] Lentz T. B. Gray S. J. Samulski R. J. (2012). Viral vectors for gene delivery to the central nervous system. Neurobiol. Dis. 48, 179–188. 10.1016/j.nbd.2011.09.014 22001604 PMC3293995

[B99] Leone P. Shera D. McPhee S. W. Francis J. S. Kolodny E. H. Bilaniuk L. T.. (2012). Long-term follow-up after gene therapy for canavan disease. Sci. Transl. Med. 4:165ra163. 10.1126/scitranslmed.3003454 23253610 PMC3794457

[B100] Levy J. M. Yeh W. H. Pendse N. Davis J. R. Hennessey E. Butcher R.. (2020). Cytosine and adenine base editing of the brain, liver, retina, heart and skeletal muscle of mice *via* adeno-associated viruses. Nat. Biomed. Eng. 4, 97–110. 10.1038/s41551-019-0501-5 31937940 PMC6980783

[B101] Lewis P. F. Emerman M. (1994). Passage through mitosis is required for oncoretroviruses but not for the human immunodeficiency virus. J. Virol. 68, 510–516. 10.1128/jvi.68.1.510-516.1994 8254763 PMC236313

[B102] Li X. Qian X. Wang B. Xia Y. Zheng Y. Du L.. (2020). Programmable base editing of mutated TERT promoter inhibits brain tumour growth. Nat. Cell Biol. 22, 282–288. 10.1038/s41556-020-0471-6 32066906

[B103] Liao H. K. Hatanaka F. Araoka T. Reddy P. Wu M. Z. Sui Y.. (2017). *in vivo* target gene activation *via* CRISPR/Cas9-mediated trans-epigenetic modulation. Cell 171, 1495.e15–1507.e15. 10.1016/j.cell.2017.10.025 29224783 PMC5732045

[B104] Lim C. K. W. Gapinske M. Brooks A. K. Woods W. S. Powell J. E. Zeballos C. M.. (2020). Treatment of a mouse model of ALS by *in vivo* base editing. Mol. Ther. 28, 1177–1189. 10.1016/j.ymthe.2020.01.005 31991108 PMC7132599

[B105] Lisowski L. Tay S. S. Alexander I. E. (2015). Adeno-associated virus serotypes for gene therapeutics. Curr. Opin. Pharmacol. 24, 59–67. 10.1016/j.coph.2015.07.006 26291407

[B106] Liu X. S. Wu H. Ji X. Stelzer Y. Wu X. Czauderna S.. (2016). Editing DNA methylation in the mammalian genome. Cell 167, 233.e17–247.e17. 10.1016/j.cell.2016.08.056 27662091 PMC5062609

[B107] Liu X. S. Wu H. Krzisch M. Wu X. Graef J. Muffat J.. (2018). Rescue of fragile X syndrome neurons by DNA methylation editing of the fMR1 gene. Cell 172, 979.e6–992.e6. 10.1016/j.cell.2018.01.012 29456084 PMC6375087

[B108] Lu-Nguyen N. B. Broadstock M. Yáñez-Muñoz R. J. (2016). Intrastriatal delivery of integration-deficient lentiviral vectors in a rat model of Parkinson’s disease. Methods Mol. Biol. 1448, 175–184. 10.1007/978-1-4939-3753-0_13 27317181

[B109] Lusby E. Fife K. H. Berns K. I. (1980). Nucleotide sequence of the inverted terminal repetition in adeno-associated virus DNA. J. Virol. 34, 402–409. 10.1128/jvi.34.2.402-409.1980 6246271 PMC288718

[B110] Lutz M. W. Sprague D. Barrera J. Chiba-Falek O. (2020). Shared genetic etiology underlying Alzheimer’s disease and major depressive disorder. Transl. Psychiatry 10:88. 10.1038/s41398-020-0769-y 32152295 PMC7062839

[B111] Maeder M. L. Linder S. J. Cascio V. M. Fu Y. Ho Q. H. Joung J. K. (2013). CRISPR RNA-guided activation of endogenous human genes. Nat. Methods 10, 977–979. 10.1038/nmeth.2598 23892898 PMC3794058

[B112] Makarova K. S. Koonin E. V. (2015). Annotation and classification of CRISPR-cas systems. Methods Mol. Biol. 1311, 47–75. 10.1007/978-1-4939-2687-9_4 25981466 PMC5901762

[B113] Makarova K. S. Wolf Y. I. Alkhnbashi O. S. Costa F. Shah S. A. Saunders S. J.. (2015). An updated evolutionary classification of CRISPR-Cas systems. Nat. Rev. Microbiol. 13, 722–736. 10.1038/nrmicro3569 26411297 PMC5426118

[B114] Mandel R. J. Burger C. (2004). Clinical trials in neurological disorders using AAV vectors: promises and challenges. Curr. Opin. Mol. Ther. 6, 482–490. 15537049

[B115] Masters C. L. Bateman R. Blennow K. Rowe C. C. Sperling R. A. Cummings J. L. (2015). Alzheimer’s disease. Nat. Rev. Dis. Primers 1:15056. 10.1038/nrdp.2015.56 27188934

[B116] McCarty D. M. Young S. M. Jr. Samulski R. J. (2004). Integration of adeno-associated virus (AAV) and recombinant AAV vectors. Annu. Rev. Genet. 38, 819–845. 10.1146/annurev.genet.37.110801.143717 15568995

[B117] McCormack A. L. Mak S. K. Henderson J. M. Bumcrot D. Farrer M. J. Di Monte D. A. (2010). α-synuclein suppression by targeted small interfering RNA in the primate substantia nigra. PLoS One 5:e12122. 10.1371/journal.pone.0012122 20711464 PMC2920329

[B118] McDonald J. I. Celik H. Rois L. E. Fishberger G. Fowler T. Rees R.. (2016). Reprogrammable CRISPR/Cas9-based system for inducing site-specific DNA methylation. Biol. Open 5, 866–874. 10.1242/bio.019067 27170255 PMC4920199

[B119] McIntosh A. M. Bennett C. Dickson D. Anestis S. F. Watts D. P. Webster T. H.. (2012). The apolipoprotein E (APOE) gene appears functionally monomorphic in chimpanzees (*Pan troglodytes*). PLoS One 7:e47760. 10.1371/journal.pone.0047760 23112842 PMC3480407

[B120] Miller D. G. Adam M. A. Miller A. D. (1990). Gene transfer by retrovirus vectors occurs only in cells that are actively replicating at the time of infection. Mol. Cell. Biol. 10, 4239–4242. 10.1128/mcb.10.8.4239 2370865 PMC360961

[B121] Mitchell A. M. Nicolson S. C. Warischalk J. K. Samulski R. J. (2010). AAV’s anatomy: roadmap for optimizing vectors for translational success. Curr. Gene Ther. 10, 319–340. 10.2174/156652310793180706 20712583 PMC3920455

[B122] Montini E. Cesana D. Schmidt M. Sanvito F. Bartholomae C. C. Ranzani M.. (2009). The genotoxic potential of retroviral vectors is strongly modulated by vector design and integration site selection in a mouse model of HSC gene therapy. J. Clin. Invest. 119, 964–975. 10.1172/JCI37630 19307726 PMC2662564

[B123] Montini E. Cesana D. Schmidt M. Sanvito F. Ponzoni M. Bartholomae C.. (2006). Hematopoietic stem cell gene transfer in a tumor-prone mouse model uncovers low genotoxicity of lentiviral vector integration. Nat. Biotechnol. 24, 687–696. 10.1038/nbt1216 16732270

[B124] Moreno A. M. Fu X. Zhu J. Katrekar D. Shih Y. V. Marlett J.. (2018). In situ gene therapy *via* AAV-CRISPR-Cas9-mediated targeted gene regulation. Mol. Ther. 26, 1818–1827. 10.1016/j.ymthe.2018.04.017 29754775 PMC6035733

[B125] Morita S. Noguchi H. Horii T. Nakabayashi K. Kimura M. Okamura K.. (2016). Targeted DNA demethylation *in vivo* using dCas9-peptide repeat and scFv-TET1 catalytic domain fusions. Nat. Biotechnol. 34, 1060–1065. 10.1038/nbt.3658 27571369

[B126] Moskvina V. Harold D. Russo G. Vedernikov A. Sharma M. Saad M.. (2013). Analysis of genome-wide association studies of Alzheimer disease and of Parkinson disease to determine if these 2 diseases share a common genetic risk. JAMA Neurol. 70, 1268–1276. 10.1001/jamaneurol.2013.448 23921447 PMC5978422

[B127] Mueller K. Carlson-Stevermer J. Saha K. (2018). Increasing the precision of gene editing *in vitro*, *ex vivo*, and *in vivo*. Curr. Opin. Biomed. Eng. 7, 83–90. 10.1016/j.cobme.2018.08.006 31086832 PMC6510258

[B128] Mullard A. (2019). Anti-amyloid failures stack up as Alzheimer antibody flops. Nat. Rev. Drug Discov. [Epub ahead of print]. 10.1038/d41573-019-00064-1 31048802

[B129] Naldini L. Blomer U. Gage F. H. Trono D. Verma I. M. (1996). Efficient transfer, integration and sustained long-term expression of the transgene in adult rat brains injected with a lentiviral vector. Proc. Natl. Acad. Sci. U S A 93, 11382–11388. 10.1073/pnas.93.21.11382 8876144 PMC38066

[B130] Nash K. Chen W. Muzyczka N. (2008). Complete *in vitro* reconstitution of adeno-associated virus DNA replication requires the minichromosome maintenance complex proteins. J. Virol. 82, 1458–1464. 10.1128/JVI.01968-07 18057257 PMC2224442

[B131] Niemeyer G. P. Herzog R. W. Mount J. Arruda V. R. Tillson D. M. Hathcock J.. (2009). Long-term correction of inhibitor-prone hemophilia B dogs treated with liver-directed AAV2-mediated factor IX gene therapy. Blood 113, 797–806. 10.1182/blood-2008-10-181479 18957684 PMC2630266

[B132] Nishimasu H. Shi X. Ishiguro S. Gao L. Hirano S. Okazaki S.. (2018). Engineered CRISPR-Cas9 nuclease with expanded targeting space. Science 361, 1259–1262. 10.1126/science.aas9129 30166441 PMC6368452

[B133] Nussbaum R. L. (2013). Genome-wide association studies, Alzheimer disease and understudied populations. JAMA 309, 1527–1528. 10.1001/jama.2013.3507 23571593

[B134] Nussbaum R. L. (2018). Genetics of synucleinopathies. Cold Spring Harb. Perspect. Med. 8:a024109. 10.1101/cshperspect.a024109 28213435 PMC5983162

[B135] O’Geen H. Ren C. Nicolet C. M. Perez A. A. Halmai J. Le V. M.. (2017). dCas9-based epigenome editing suggests acquisition of histone methylation is not sufficient for target gene repression. Nucleic Acids Res. 45, 9901–9916. 10.1093/nar/gkx578 28973434 PMC5622328

[B136] Ortinski P. I. O’Donovan B. Dong X. Kantor B. (2017). Integrase-deficient lentiviral vector as an all-in-one platform for highly efficient CRISPR/Cas9-mediated gene editing. Mol. Ther. Methods Clin. Dev. 5, 153–164. 10.1016/j.omtm.2017.04.002 28497073 PMC5424571

[B137] Pal S. (2012). Selected neurodegenerative disorders. US Pharm. 37:6 Available online at: https://www.uspharmacist.com/article/selected-neurodegenerative-disorders.

[B138] Park H. Oh J. Shim G. Cho B. Chang Y. Kim S.. (2019). *in vivo* neuronal gene editing *via* CRISPR-Cas9 amphiphilic nanocomplexes alleviates deficits in mouse models of Alzheimer’s disease. Nat. Neurosci. 22, 524–528. 10.1038/s41593-019-0352-0 30858603

[B139] Perez-Pinera P. Kocak D. D. Vockley C. M. Adler A. F. Kabadi A. M. Polstein L. R.. (2013). RNA-guided gene activation by CRISPR-Cas9-based transcription factors. Nat. Methods 10, 973–976. 10.1038/nmeth.2600 23892895 PMC3911785

[B140] Pflueger C. Tan D. Swain T. Nguyen T. Pflueger J. Nefzger C.. (2018). A modular dCas9-SunTag DNMT3A epigenome editing system overcomes pervasive off-target activity of direct fusion dCas9-DNMT3A constructs. Genome Res. 28, 1193–1206. 10.1101/gr.233049.117 29907613 PMC6071642

[B141] Philippe S. Sarkis C. Barkats M. Mammeri H. Ladroue C. Petit C.. (2006). Lentiviral vectors with a defective integrase allow efficient and sustained transgene expression *in vitro* and *in vivo*. Proc. Natl. Acad. Sci. U S A 103, 17684–17689. 10.1073/pnas.0606197103 17095605 PMC1693807

[B142] Pickar-Oliver A. Gersbach C. A. (2019). The next generation of CRISPR-Cas technologies and applications. Nat. Rev. Mol. Cell Biol. 20, 490–507. 10.1038/s41580-019-0131-5 31147612 PMC7079207

[B143] Pitas R. E. Boyles J. K. Lee S. H. Hui D. Weisgraber K. H. (1987). Lipoproteins and their receptors in the central nervous system. Characterization of the lipoproteins in cerebrospinal fluid and identification of apolipoprotein B,E(LDL) receptors in the brain. J. Biol. Chem. 262, 14352–14360. 3115992

[B144] Poewe W. Seppi K. Tanner C. M. Halliday G. M. Brundin P. Volkmann J.. (2017). Parkinson disease. Nat. Rev. Dis. Primers 3:17013. 10.1038/nrdp.2017.13 28332488

[B145] Powell S. K. Khan N. Parker C. L. Samulski R. J. Matsushima G. Gray S. J.. (2016). Characterization of a novel adeno-associated viral vector with preferential oligodendrocyte tropism. Gene Ther. 23, 807–814. 10.1038/gt.2016.62 27628693 PMC5541369

[B146] Qi L. S. Larson M. H. Gilbert L. A. Doudna J. A. Weissman J. S. Arkin A. P.. (2013). Repurposing CRISPR as an RNA-guided platform for sequence-specific control of gene expression. Cell 152, 1173–1183. 10.1016/j.cell.2013.02.022 23452860 PMC3664290

[B147] Rauch S. He E. Srienc M. Zhou H. Zhang Z. Dickinson B. C. (2019). Programmable RNA-guided RNA effector proteins built from human parts. Cell 178, 122.e12–134.e12. 10.1016/j.cell.2019.05.049 31230714 PMC6657360

[B148] Rees H. A. Liu D. R. (2018). Base editing: precision chemistry on the genome and transcriptome of living cells. Nat. Rev. Genet. 19, 770–788. 10.1038/s41576-018-0059-1 30323312 PMC6535181

[B149] Richter M. F. Zhao K. T. Eton E. Lapinaite A. Newby G. A. Thuronyi B. W.. (2020). Phage-assisted evolution of an adenine base editor with improved Cas domain compatibility and activity. Nat. Biotechnol. 38:901. 10.1038/s41587-020-0562-8 32433548

[B150] Rivera V. M. Gao G. P. Grant R. L. Schnell M. A. Zoltick P. W. Rozamus L. W.. (2005). Long-term pharmacologically regulated expression of erythropoietin in primates following AAV-mediated gene transfer. Blood 105, 1424–1430. 10.1182/blood-2004-06-2501 15507527

[B151] Rosario A. M. Cruz P. E. Ceballos-Diaz C. Strickland M. R. Siemienski Z. Pardo M.. (2016). Microglia-specific targeting by novel capsid-modified AAV6 vectors. Mol. Ther. Methods Clin. Dev. 3:16026. 10.1038/mtm.2016.26 27308302 PMC4909093

[B152] Ryu S. M. Koo T. Kim K. Lim K. Baek G. Kim S. T.. (2018). Adenine base editing in mouse embryos and an adult mouse model of Duchenne muscular dystrophy. Nat. Biotechnol. 36, 536–539. 10.1038/nbt.4148 29702637

[B153] Saida H. Matsuzaki Y. Takayama K. Iizuka A. Konno A. Yanagi S.. (2014). One-year follow-up of transgene expression by integrase-defective lentiviral vectors and their therapeutic potential in spinocerebellar ataxia model mice. Gene Ther. 21, 820–827. 10.1038/gt.2014.60 24989813

[B154] Samaranch L. Salegio E. A. San Sebastian W. Kells A. P. Foust K. D. Bringas J. R.. (2012). Adeno-associated virus serotype 9 transduction in the central nervous system of nonhuman primates. Hum. Gene Ther. 23, 382–389. 10.1089/hum.2011.200 22201473 PMC3327605

[B155] Samulski R. J. Zhu X. Xiao X. Brook J. D. Housman D. E. Epstein N.. (1991). Targeted integration of adeno-associated virus (AAV) into human chromosome 19. EMBO J. 10, 3941–3950. 1657596 10.1002/j.1460-2075.1991.tb04964.x PMC453134

[B156] Saunderson E. A. Stepper P. Gomm J. J. Hoa L. Morgan A. Allen M. D.. (2017). Hit-and-run epigenetic editing prevents senescence entry in primary breast cells from healthy donors. Nat. Commun. 8:1450. 10.1038/s41467-017-01078-2 29133799 PMC5684409

[B157] Schnepp B. C. Jensen R. L. Chen C. L. Johnson P. R. Clark K. R. (2005). Characterization of adeno-associated virus genomes isolated from human tissues. J. Virol. 79, 14793–14803. 10.1128/jvi.79.23.14793-14803.2005 16282479 PMC1287572

[B158] Scott L. Dawson V. L. Dawson T. M. (2017). Trumping neurodegeneration: targeting common pathways regulated by autosomal recessive Parkinson’s disease genes. Exp. Neurol. 298, 191–201. 10.1016/j.expneurol.2017.04.008 28445716 PMC5653467

[B159] Shmakov S. Smargon A. Scott D. Cox D. Pyzocha N. Yan W.. (2017). Diversity and evolution of class 2 CRISPR-Cas systems. Nat. Rev. Microbiol. 15, 169–182. 10.1038/nrmicro.2016.184 28111461 PMC5851899

[B160] Shore B. Shore V. (1974). An apolipoprotein preferentially enriched in cholesteryl ester-rich very low density lipoproteins. Biochem. Biophys. Res. Commun. 58, 1–7. 10.1016/0006-291x(74)90882-1 4364615

[B161] Slaymaker I. M. Gao L. Zetsche B. Scott D. A. Yan W. X. Zhang F. (2016). Rationally engineered Cas9 nucleases with improved specificity. Science 351, 84–88. 10.1126/science.aad5227 26628643 PMC4714946

[B162] Smith R. H. (2008). Adeno-associated virus integration: virus versus vector. Gene Ther. 15, 817–822. 10.1038/gt.2008.55 18401436

[B163] Sonntag F. Schmidt K. Kleinschmidt J. A. (2010). A viral assembly factor promotes AAV2 capsid formation in the nucleolus. Proc. Natl. Acad. Sci. U S A 107, 10220–10225. 10.1073/pnas.1001673107 20479244 PMC2890453

[B164] Sorek R. Kunin V. Hugenholtz P. (2008). CRISPR–a widespread system that provides acquired resistance against phages in bacteria and archaea. Nat. Rev. Microbiol. 6, 181–186. 10.1038/nrmicro1793 18157154

[B165] Srivastava A. Lusby E. W. Berns K. I. (1983). Nucleotide sequence and organization of the adeno-associated virus 2 genome. J. Virol. 45, 555–564. 10.1128/jvi.45.2.555-564.1983 6300419 PMC256449

[B166] Stepper P. Kungulovski G. Jurkowska R. Z. Chandra T. Krueger F. Reinhardt R.. (2017). Efficient targeted DNA methylation with chimeric dCas9-Dnmt3a-Dnmt3L methyltransferase. Nucleic Acids Res. 45, 1703–1713. 10.1093/nar/gkw1112 27899645 PMC5389507

[B168] Summerford C. Bartlett J. S. Samulski R. J. (1999). αVβ5 integrin: a co-receptor for adeno-associated virus type 2 infection. Nat. Med. 5, 78–82. 10.1038/4768 9883843

[B167] Summerford C. Samulski R. J. (1998). Membrane-associated heparan sulfate proteoglycan is a receptor for adeno-associated virus type 2 virions. J. Virol. 72, 1438–1445. 10.1128/jvi.72.2.1438-1445.1998 9445046 PMC124624

[B169] Tagliafierro L. Chiba-Falek O. (2016). Up-regulation of SNCA gene expression: implications to synucleinopathies. Neurogenetics 17, 145–157. 10.1007/s10048-016-0478-0 26948950 PMC4907864

[B170] Takahashi M. Suzuki M. Fukuoka M. Fujikake N. Watanabe S. Murata M.. (2015). Normalization of overexpressed α-synuclein causing Parkinson’s disease by a moderate gene silencing with RNA interference. Mol. Ther. Nucleic Acids 4:e241. 10.1038/mtna.2015.14 25965551

[B171] Tervo D. G. Hwang B. Y. Viswanathan S. Gaj T. Lavzin M. Ritola K. D.. (2016). A designer AAV variant permits efficient retrograde access to projection neurons. Neuron 92, 372–382. 10.1016/j.neuron.2016.09.021 27720486 PMC5872824

[B172] Thakore P. I. Black J. B. Hilton I. B. Gersbach C. A. (2016). Editing the epigenome: technologies for programmable transcription and epigenetic modulation. Nat. Methods 13, 127–137. 10.1038/nmeth.3733 26820547 PMC4922638

[B173] Thakore P. I. D’Ippolito A. M. Song L. Safi A. Shivakumar N. K. Kabadi A. M.. (2015). Highly specific epigenome editing by CRISPR-Cas9 repressors for silencing of distal regulatory elements. Nat. Methods 12, 1143–1149. 10.1038/nmeth.3630 26501517 PMC4666778

[B174] Thakore P. I. Kwon J. B. Nelson C. E. Rouse D. C. Gemberling M. P. Oliver M. L.. (2018). RNA-guided transcriptional silencing *in vivo* with S. aureus CRISPR-Cas9 repressors. Nat. Commun. 9:1674. 10.1038/s41467-018-04048-4 29700298 PMC5920046

[B175] Themis M. Waddington S. N. Schmidt M. von Kalle C. Wang Y. Al-Allaf F.. (2005). Oncogenesis following delivery of a nonprimate lentiviral gene therapy vector to fetal and neonatal mice. Mol. Ther. 12, 763–771. 10.1016/j.ymthe.2005.07.358 16084128

[B176] van Haasteren J. Li J. Scheideler O. J. Murthy N. Schaffer D. V. (2020). The delivery challenge: fulfilling the promise of therapeutic genome editing. Nat. Biotechnol. 38, 845–855. 10.1038/s41587-020-0565-5 32601435

[B177] Vijayraghavan S. Kantor B. (2017). A protocol for the production of integrase-deficient lentiviral vectors for CRISPR/Cas9-mediated gene knockout in dividing cells. J. Vis. Exp. 130:56915. 10.3791/56915 29286484 PMC5755554

[B178] Vojta A. Dobrinic P. Tadic V. Bockor L. Korac P. Julg B.. (2016). Repurposing the CRISPR-Cas9 system for targeted DNA methylation. Nucleic Acids Res. 44, 5615–5628. 10.1093/nar/gkw159 26969735 PMC4937303

[B179] Walton R. T. Christie K. A. Whittaker M. N. Kleinstiver B. P. (2020). Unconstrained genome targeting with near-PAMless engineered CRISPR-Cas9 variants. Science 368, 290–296. 10.1126/science.aba8853 32217751 PMC7297043

[B180] Wang C. Najm R. Xu Q. Jeong D. E. Walker D. Balestra M. E.. (2018). Gain of toxic apolipoprotein E4 effects in human iPSC-derived neurons is ameliorated by a small-molecule structure corrector. Nat. Med. 24, 647–657. 10.1038/s41591-018-0004-z 29632371 PMC5948154

[B181] Wang D. Zhang F. Gao G. (2020). CRISPR-based therapeutic genome editing: strategies and *in vivo* delivery by AAV vectors. Cell 181, 136–150. 10.1016/j.cell.2020.03.023 32243786 PMC7236621

[B182] Wangensteen K. J. Wang Y. J. Dou Z. Wang A. W. Mosleh-Shirazi E. Horlbeck M. A.. (2018). Combinatorial genetics in liver repopulation and carcinogenesis with a *in vivo* CRISPR activation platform. Hepatology 68, 663–676. 10.1002/hep.29626 29091290 PMC5930141

[B183] Weltner J. Balboa D. Katayama S. Bespalov M. Krjutskov K. Jouhilahti E. M.. (2018). Human pluripotent reprogramming with CRISPR activators. Nat. Commun. 9:2643. 10.1038/s41467-018-05067-x 29980666 PMC6035213

[B184] Wu Z. Asokan A. Samulski R. J. (2006). Adeno-associated virus serotypes: vector toolkit for human gene therapy. Mol. Ther. 14, 316–327. 10.1016/j.ymthe.2006.05.009 16824801

[B185] Xiao X. Li J. Samulski R. J. (1998). Production of high-titer recombinant adeno-associated virus vectors in the absence of helper adenovirus. J. Virol. 72, 2224–2232. 10.1128/jvi.72.3.2224-2232.1998 9499080 PMC109519

[B186] Yáñez-Muñoz R. J. Balaggan K. S. MacNeil A. Howe S. J. Schmidt M. Smith A. J.. (2006). Effective gene therapy with nonintegrating lentiviral vectors. Nat. Med. 12, 348–353. 10.1038/nm1365 16491086

[B187] Yeh W. H. Chiang H. Rees H. A. Edge A. S. B. Liu D. R. (2018). *in vivo* base editing of post-mitotic sensory cells. Nat. Commun. 9:2184. 10.1038/s41467-018-04580-3 29872041 PMC5988727

[B188] Yeo N. C. Chavez A. Lance-Byrne A. Chan Y. Menn D. Milanova D.. (2018). An enhanced CRISPR repressor for targeted mammalian gene regulation. Nat. Methods 15, 611–616. 10.1038/s41592-018-0048-5 30013045 PMC6129399

[B189] Yin H. Kauffman K. J. Anderson D. G. (2017). Delivery technologies for genome editing. Nat. Rev. Drug Discov. 16, 387–399. 10.1038/nrd.2016.280 28337020

[B190] Zennou V. Petit C. Guetard D. Nerhbass U. Montagnier L. Charneau P. (2000). HIV-1 genome nuclear import is mediated by a central DNA flap. Cell 101, 173–185. 10.1016/s0092-8674(00)80828-4 10786833

[B191] Zheng Y. Shen W. Zhang J. Yang B. Liu Y. N. Qi H.. (2018). CRISPR interference-based specific and efficient gene inactivation in the brain. Nat. Neurosci. 21, 447–454. 10.1038/s41593-018-0077-5 29403034

[B192] Zhou H. Liu J. Zhou C. Gao N. Rao Z. Li H.. (2018). *In vivo* simultaneous transcriptional activation of multiple genes in the brain using CRISPR-dCas9-activator transgenic mice. Nat. Neurosci. 21, 440–446. 10.1038/s41593-017-0060-6 29335603

[B193] Zufferey R. Donello J. E. Trono D. Hope T. J. (1999). Woodchuck hepatitis virus posttranscriptional regulatory element enhances expression of transgenes delivered by retroviral vectors. J. Virol. 73, 2886–2892. 10.1128/jvi.73.4.2886-2892.1999 10074136 PMC104046

[B194] Zufferey R. Nagy D. Mandel R. J. Naldini L. Trono D. (1997). Multiply attenuated lentiviral vector achieves efficient gene delivery *in vivo*. Nat. Biotechnol. 15, 871–875. 10.1038/nbt0997-871 9306402

